# Ion-pair cooperativity in common neutral salts promotes methanolysis of ester-containing plastics

**DOI:** 10.1039/d6sc04874a

**Published:** 2026-09-15

**Authors:** Yushuang Huang, Junjian Xie, Jiawei Xie, Yisong Zhou, Yuan Lei, Tinghao Jia, Jinjin Li, Chang-An Zhou, Chao Wang, Lei Song, Kui Ma, Hairong Yue, Ji-Jun Zou

**Affiliations:** a Institute of New Energy and Low-Carbon Technology, Sichuan University Chengdu 610207 China xiejiawei@scu.edu.cn; b College of Materials and Chemistry and Chemical Engineering, Chengdu University of Technology Chengdu 610059 China; c School of Chemical Engineering, Sichuan University Chengdu 610065 China; d School of Chemistry and Chemical Engineering, Northwestern Polytechnical University Xi'an 710072 China; e State Key Laboratory of Advanced Polymer Materials (Sichuan University), Sichuan University Chengdu 610065 China; f College of Chemical and Biological Engineering, Zhejiang University Hangzhou 310027 China; g Research Institute of Natural Gas Technology, PetroChina Southwest Oil & Gasfield Company Chengdu 610299 China; h School of Chemical Engineering and Technology, Tianjin University Tianjin 300072 China

## Abstract

Methanolysis is a promising strategy for chemical recycling of ester-containing plastics, but current approaches rely on homogeneous acids/bases or heterogeneous catalysts, which suffer from corrosion and deactivation. Here we report that common neutral salts can promote plastic methanolysis *via* an ion-pair cooperative mechanism. *In situ* spectroscopy, nuclear magnetic resonance, Raman spectroscopy, molecular dynamics simulations, and density functional theory calculations reveal that Na^+^ coordinates with carbonyl oxygen, enhancing carbonyl electrophilicity, while Cl^−^ interacts with methanol through O–H⋯Cl^−^ hydrogen bonding, disrupting its hydrogen-bond network and activating the nucleophile. Extending this concept to various neutral salts establishes a clear structure–activity relationship governed by cation-mediated substrate activation and anion-mediated methanol activation. Notably, naturally occurring salts also show comparable activity, and the strategy is applicable to representative ester-containing plastics including polycarbonate (PC), polylactic acid (PLA), polybutylene terephthalate (PBT), and polyethylene terephthalate (PET). These results identify ion-pair cooperativity as a possible mechanism for polymer depolymerization and provide a feasible strategy for plastic chemical recycling.

## Abstract 2

Ion-pair cooperativity of common neutral salts promotes methanolysis of polycarbonate, polylactic acid, polybutylene terephthalate, and polyethylene terephthalate.

## Graphical abstract

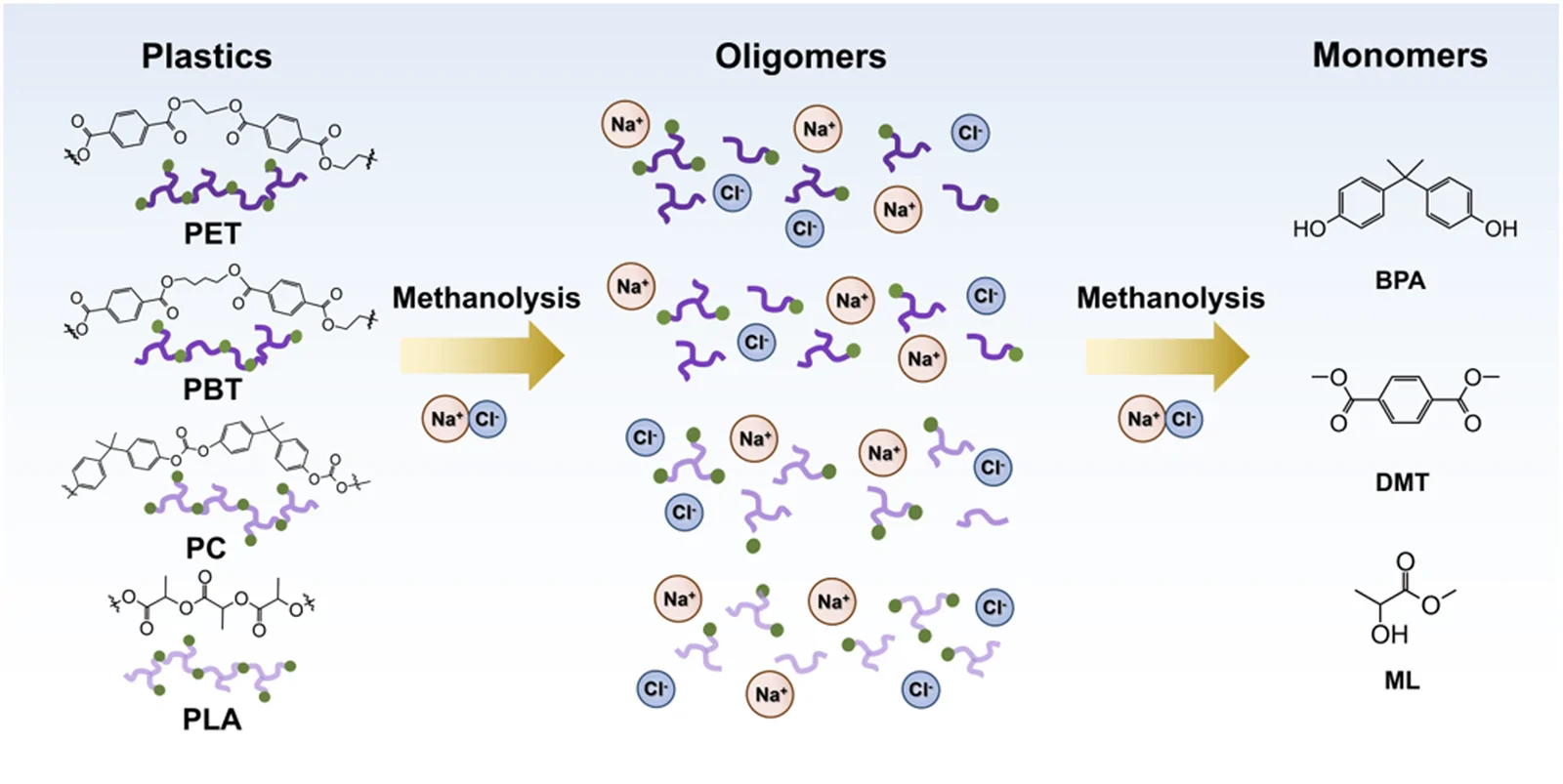

## Introduction

Since 1950, the rapid expansion of plastic production has been accompanied by a parallel increase in plastic waste generation and mismanagement. In 2020, global annual plastic consumption had exceeded 500 million metric tons.^1^ Among the various classes of plastics, ester-containing plastics such as polyethylene terephthalate (PET), polybutylene terephthalate (PBT), polycarbonates (PC), and polylactic acid (PLA) represent the most widely produced commodity polymers, with extensive applications in packaging, textiles, and the electronics industry.^2,3^ The continuous growth in ester-containing plastic production has inevitably led to a substantial increase in plastic waste. However, the majority of this waste is still treated using conventional disposal methods, including landfilling, incineration, or direct release into the marine environment.^4–6^ These practices not only exacerbate plastic pollution and greenhouse gas emissions, but also result in the irreversible loss of valuable carbon resources. Therefore, the development of efficient and sustainable strategies for the recycling and valorization of plastic waste has become an urgent and widely recognized research priority.

Compared with mechanical recycling, which typically suffers from low efficiency and limited product quality, chemical recycling enables plastics to be converted back into their constituent monomers for repolymerization into virgin materials or transformed into high-value chemicals.^7–14^ In recent years, methanolysis has emerged as one of the most promising strategies for chemical recycling.^15–19^ Catalytic systems developed for this process can be broadly classified into homogeneous bases (*e.g.*, NaOH, K_2_CO_3_, and organic bases),^20–24^ heterogeneous solid catalysts (*e.g.*, alkaline earth metal oxides, layered double oxides, and supported catalysts),^25–27^ and emerging metal-free systems (*e.g.*, ionic liquids, deep eutectic solvents, and polymeric carbon nitride).^28–31^ Among these, heterogeneous catalysts^25,26^ such as Mg/Al layered double oxides, CaO or CeO_2_ modified mesoporous materials, and composite oxides with engineered interfaces have demonstrated outstanding performance, frequently achieving nearly quantitative PC conversion and BPA yields above 95% under relatively mild conditions (60–130 °C). Mechanistic studies^20–32^ suggest that interfacial synergistic effects, oxygen vacancies, and strong basic sites play key roles in activating methanol, primarily through methoxide generation, which is widely recognized as the critical step. In addition, structural engineering strategies,^25–27,33,34^ such as constructing hollow or mesoporous architectures, have been shown to enhance active site accessibility, reduce diffusion limitations, and lower activation energy barriers.

Despite significant advances, different classes of catalysts still exhibit distinct advantages and inherent limitations. Homogeneous alkaline catalysts generally show high activity and fast reaction rates but suffer from drawbacks such as equipment corrosion and associated environmental concerns.^35^ In contrast, heterogeneous catalytic systems are easier to separate and recycle, making them attractive. Nevertheless, their performance often relies on the use of co-solvents to enhance polymer solubility and reaction efficiency, and they still face challenges related to the limited scalability. Notably, in recent years, neutral inorganic salts have been reported to promote PET depolymerization by hydrolysis.^36^ These studies highlighted the role of metal cations in activating the ester bond toward hydrolysis. Inspired by these findings, it was hypothesized that common neutral salts may also promote the methanolysis of ester-containing plastics.

**Fig. 1:**
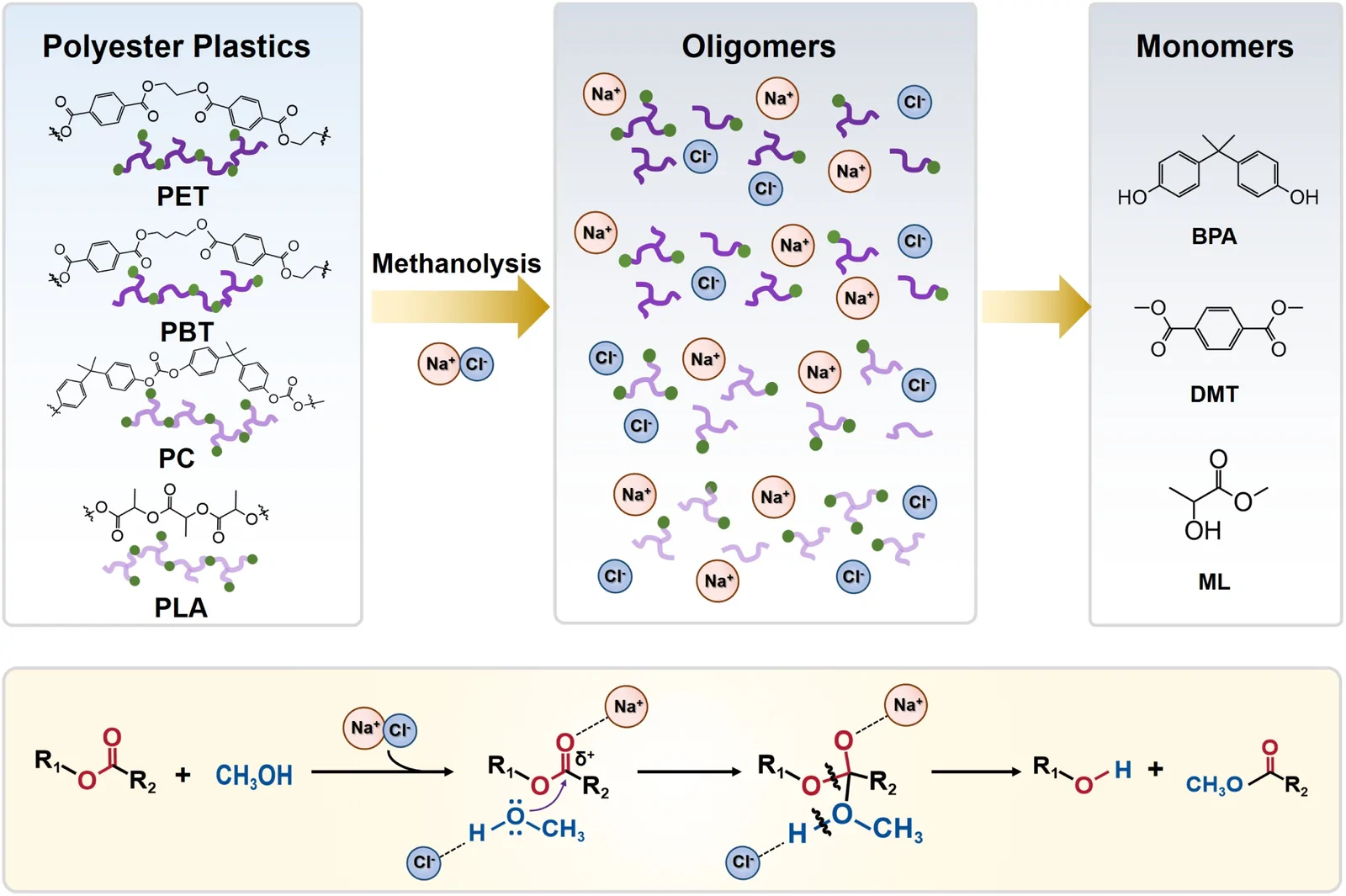
Ester-containing plastic methanolysis promoted by NaCl ion-pair cooperativity.

In this work, a common neutral salt, NaCl, was employed to enhance the methanolysis of ester-containing plastics (Fig. 1). The mechanistic role of NaCl was elucidated by combining *in situ* diffuse reflectance infrared Fourier transform spectroscopy (*in situ* DRIFTS), nuclear magnetic resonance (NMR), Raman spectroscopy, molecular dynamics (MD) simulations, and density functional theory (DFT) calculations. In addition, a series of salts with different cations and anions were investigated to establish structure–activity relationships governing salt-promoted methanolysis. The general applicability of the proposed strategy was further examined using representative ester-containing plastics, including PC, PET, PBT and PLA, providing a facile strategy for chemical recycling of ester-containing plastics.

## Results and discussion

### Methanolysis process promoted by NaCl

**Fig. 2:**
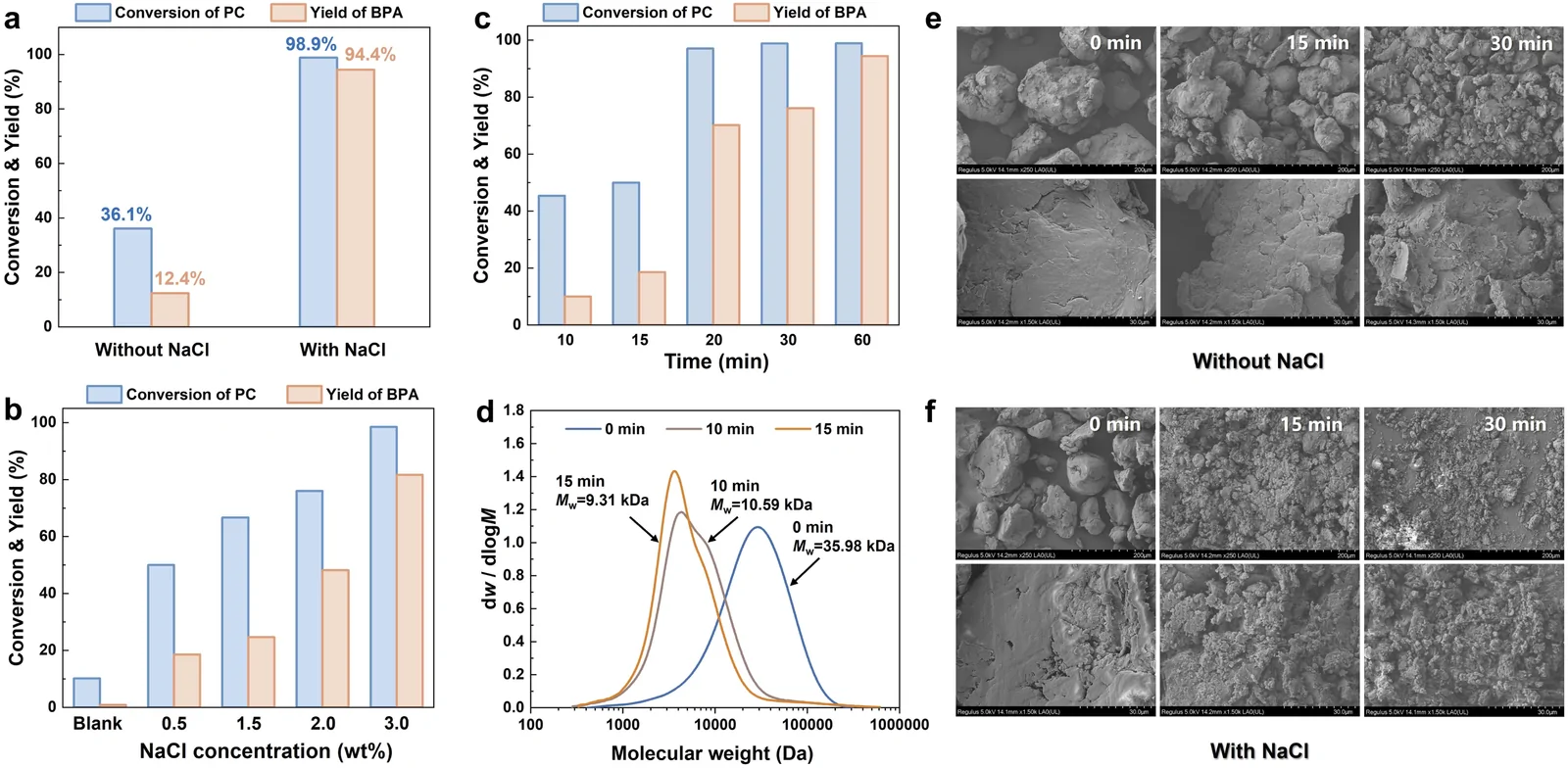
PC methanolysis process promoted by NaCl. (a) PC conversion and BPA yield in the absence and presence of NaCl. Reaction conditions: 423 K, 0.5 MPa N_2_, 60 min, 1.00 g PC powder, 0.15 g NaCl, 30.00 g methanol; (b) PC methanolysis performance over different NaCl concentrations. Reaction conditions: 423 K, 0.5 MPa N_2_, 15 min, 1.00 g PC powder, 0–0.90 g NaCl, and 30.00 g methanol; (c) time-dependent profiles of PC conversion and BPA yield in the presence of NaCl. Reaction conditions: 423 K, 0.5 MPa N_2_, 1.00 g PC powder, 0.15 g NaCl, and 30.00 g methanol; (d) GPC profiles of different reaction times over PC depolymerization. Reaction conditions: 423 K, 0.5 MPa N_2_, 1.00 g PC powder, 0.15 g NaCl, and 30.00 g methanol. SEM images of the residual solids after PC methanolysis (e) in the absence of NaCl and (f) in the presence of NaCl. Reaction conditions: 423 K, 0.5 MPa N_2_, 1.00 g PC powder, 0.15 g NaCl, and 30.00 g methanol.

To elucidate the promoting role of NaCl in the reaction, we chose PC as a model to conduct comparative methanolysis experiments in the absence and presence of NaCl. The introduction of NaCl significantly enhanced both PC conversion and BPA yield. Specifically, after 1 h of reaction, the PC conversion increased from 36.1% in the absence of NaCl to 98.9% in its presence, while the BPA yield increased from 12.4% to 94.4% (Fig. 2a). These results clearly demonstrate the strong promoting effect of NaCl on PC methanolysis. On this basis, the influence of NaCl concentration on methanolysis performance was further investigated. The results show that the efficiency of PC methanolysis improved markedly with increasing NaCl amount from 0.5 wt% to 3.0 wt%. When the NaCl concentration reached 3.0 wt% relative to methanol, the PC conversion and BPA yield reached 98.5% and 81.7%, respectively (Fig. 2b). Notably, with prolonged reaction time, even a low NaCl concentration of 0.5 wt% enabled nearly complete conversion of PC. Both PC conversion and BPA yield increased gradually with reaction time (Fig. 2c).

To further elucidate the structural evolution of PC during methanolysis, gel permeation chromatography (GPC) analyses were performed on the pristine PC and the residual solids collected after 10 and 15 min of reaction (Fig. 2d). The results reveal that rapid depolymerization occurred within the first 10 min, leading to the formation of a large fraction of low-molecular-weight oligomers, as evidenced by the weight-average molecular weight (*M*_w_) distributions shown in Fig. 2d. Specifically, the *M*_w_ decreased sharply from 35.98 kDa to 10.59 kDa after 10 min and further declined to 9.31 kDa after 15 min. In addition, the polydispersity index (PDI) increased from 2.36 to 2.66 as the *M*_w_ decreased (Table S1), indicating a progressive broadening of the molecular weight distribution during depolymerization. This behavior is likely attributable to the random cleavage of C–O bonds along the PC backbone.^37^ To further visualize the depolymerization process, scanning electron microscopy (SEM) was employed to characterize the residual solid particles during methanolysis. In the absence of NaCl, the PC particles remained relatively large and largely preserved their original surface morphology after 30 min of reaction (Fig. 2e), indicating limited depolymerization. In contrast, in the presence of NaCl, substantial particle fragmentation accompanied by a pronounced reduction in particle size was observed after only 15 min of reaction (Fig. 2f). These observations further confirm that NaCl significantly accelerates the structural degradation and depolymerization of PC.

### *In situ* experimental monitoring of NaCl-promoted methanolysis

According to previous studies, PC methanolysis proceeds through a typical nucleophilic substitution mechanism, in which methanol acts as a nucleophile and attacks the carbonate linkage (–O–C(=O)–O–) in the PC backbone, resulting in chain scission and depolymerization.^20–32,38,39^ To further elucidate the promoting role of NaCl, *in situ* DRIFTS was employed to monitor the reaction process. The experiments were conducted using a bubbling method under a methanol atmosphere.^40,41^ The temperature was gradually increased from 50 to 150 °C, and spectra were collected at 25 °C intervals. After reaching 150 °C, the system was maintained at this temperature for 1 h, during which spectra were recorded every 5 min.

**Fig. 3:**
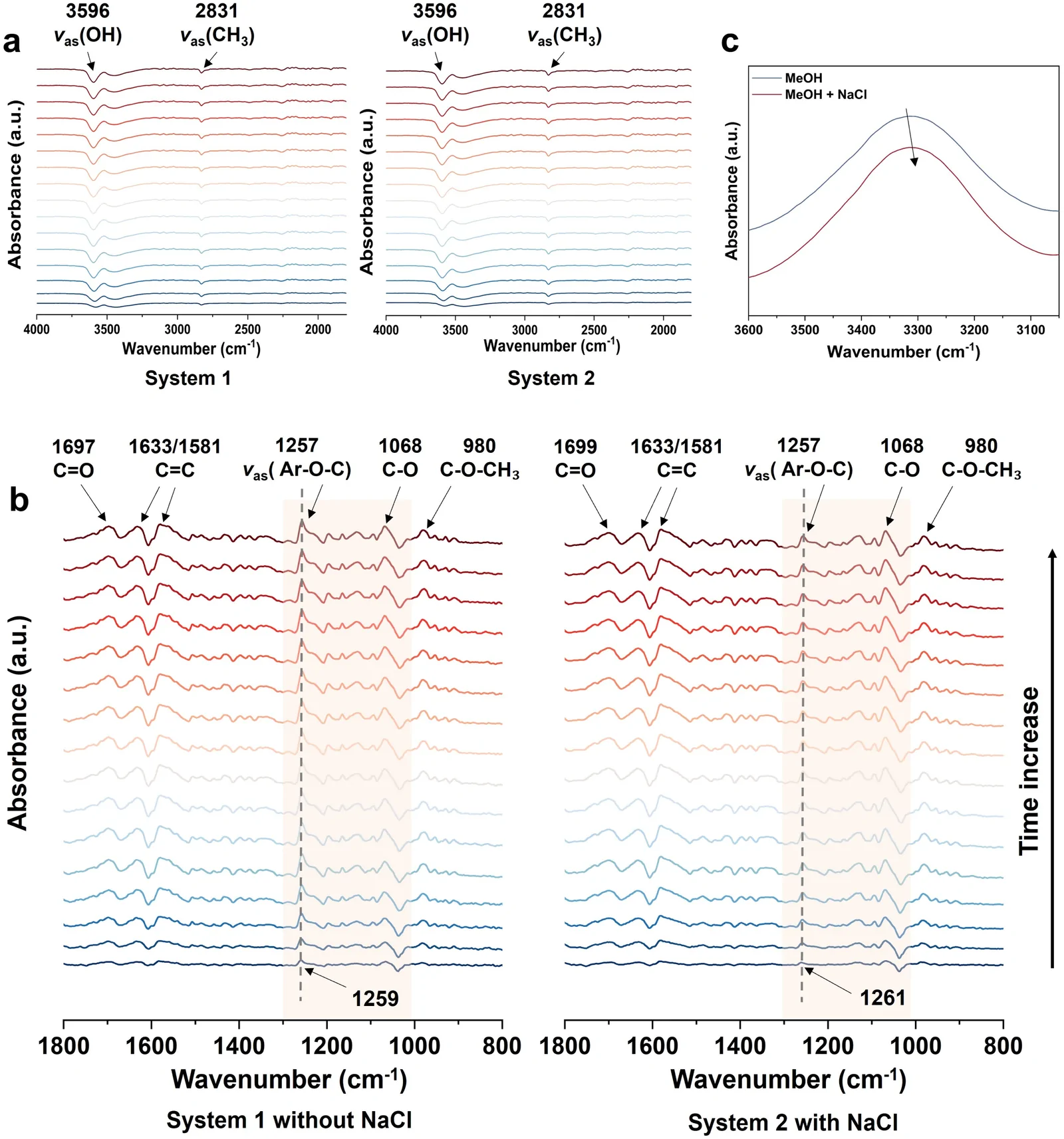
Monitoring the reaction by infrared spectroscopy. *In situ* DRIFTS spectra in (a) 4000–1800 cm^−1^ and (b) 1800–800 cm^−1^; (c) ATR-FTIR spectra of methanol with and without NaCl.

Comparison of the DRIFTS spectra for PC methanolysis in the absence of NaCl (System 1) and in the presence of NaCl (System 2) over the range of 4000–400 cm^−1^ revealed no new characteristic absorption bands throughout the reaction (Fig. 3a and b). This observation indicates that the introduction of NaCl does not alter the fundamental reaction pathway of PC methanolysis. In the 4000–1800 cm^−1^ region, two negative bands are observed at 3596 cm^−1^ (*ν*_as_(O–H)) and 2831 cm^−1^ (*ν*_as_(CH_3_)), respectively,^42–44^ and their intensities gradually increase with reaction time (Fig. 3a). This behavior arises because the background spectrum for the *in situ* DRIFTS measurements was collected after methanol adsorption had reached saturation. Therefore, the progressive growth of these negative bands reflects the continuous consumption of methanol molecules adsorbed on the PC surface, providing indirect evidence for the ongoing methanolysis reaction.

In the 1800–800 cm^−1^ region (Fig. 3b), both systems exhibit several characteristic bands, including *ν*(C=O) at ∼1700 cm^−1^, *ν*(C=C) at 1633 and 1581 cm^−1^, *ν*_as_(Ar–O–C) at 1257 cm^−1^, *ν*(C–O) at 1068 cm^−1^, and *ν*(C–O–CH_3_) at 980 cm^−1^.^45–48^ Among these, the asymmetric stretching vibration of the Ar–O–C linkage, *ν*_as_(Ar–O–C), undergoes a slight red shift, from 1259 to 1257 cm^−1^ in System 1 and from 1261 to 1257 cm^−1^ in System 2. Notably, the larger red shift observed in System 2 suggests that the Ar–O–C linkage in the PC backbone is more effectively activated in the presence of NaCl, thereby facilitating depolymerization.

In addition, clear differences are observed in the relative intensities of the *ν*_as_(Ar–O–C) band associated with PC and the *ν*(C–O) band associated with the depolymerization product BPA, highlighted in the orange shaded region in Fig. 3b. In System 1, the *ν*(C–O) band remains weaker than the *ν*_as_(Ar–O–C) band, indicating that a substantial fraction of the polymer backbone remains intact and that the extent of methanolysis is relatively limited. In contrast, System 2 exhibits the opposite trend, with the *ν*(C–O) band becoming more intense than the *ν*_as_(Ar–O–C) band, suggesting extensive cleavage of the PC backbone and significantly enhanced BPA formation. These results further confirm that NaCl accelerates the depolymerization of PC.

Furthermore, to investigate the effect of NaCl on methanol, attenuated total reflectance Fourier-transform infrared spectra (ATR-FTIR) of pure methanol and methanol containing NaCl were compared. A slight red shift in the O–H stretching vibration of methanol was observed in the presence of NaCl (Fig. 3c),^42^ indicating that NaCl not only facilitates activation of the Ar–O–C linkage in PC, but also modulates the hydrogen-bonding network of methanol, potentially enhancing its nucleophilic reactivity. In summary, these results demonstrate that the introduction of NaCl effectively promotes activation of the Ar–O–C linkage in the PC backbone while simultaneously regulating the methanol environment, thereby accelerating PC depolymerization and methanolysis.

### Theoretical investigation of ion-pair cooperative mechanism

**Fig. 4:**
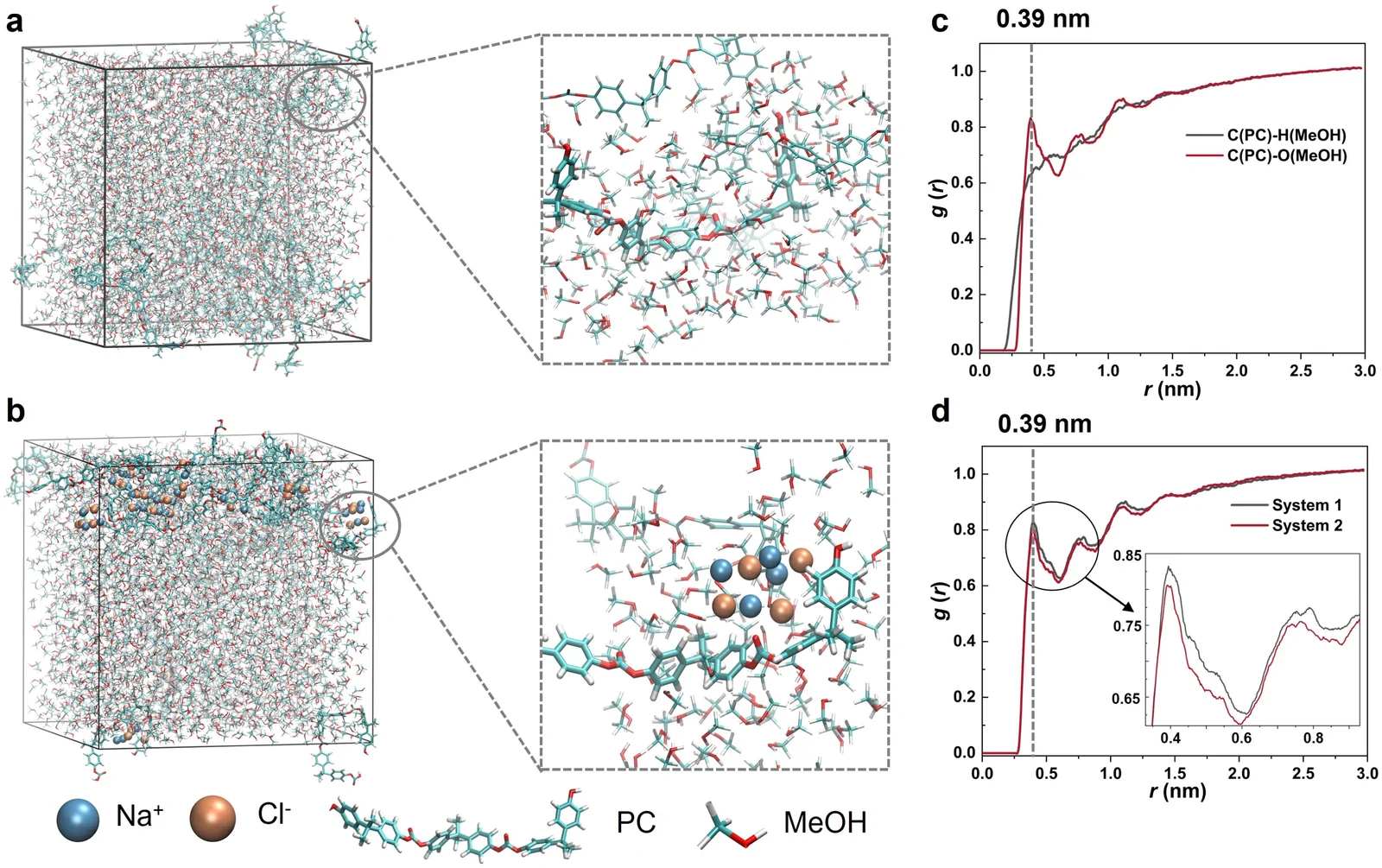
MD simulations of methanolysis in the absence and presence of NaCl. 3D snapshot of (a) System 1 and (b) System 2 obtained from MD simulations and partial enlarged snapshot representing PC solvation structure; (c) RDFs for C(PC)–O(MeOH) and C(PC)–H(MeOH) from MD simulations in System 1; (d) RDFs for C(PC)–O(MeOH) obtained from MD simulations in System 1 and System 2.

To further elucidate the effect of NaCl on the local environment surrounding PC at the molecular level and clarify its mechanistic role from a microscopic structural perspective, MD simulations were performed.^48,49^ Snapshots of the simulation boxes show that, in the absence of NaCl (System 1), the PC chains are well solvated by methanol molecules (Fig. 4a). In contrast, upon the addition of NaCl (System 2), Na^+^ and Cl^−^ preferentially accumulate in the vicinity of the PC chains, partially displacing methanol molecules from the local solvation shell (Fig. 4b). These observations indicate that the introduction of NaCl significantly modifies the local solvation structure around PC, which may consequently influence intermolecular interactions during the reaction process.

To gain deeper insight into the solvent structural changes induced by NaCl addition, radial distribution functions (RDFs) were calculated between the carbonyl carbon of PC (denoted as C(PC)) and surrounding atoms in both systems.^49–51^ In System 1, a pronounced RDF peak appears at approximately 3.9 Å for the C(PC)–O(MeOH) pair, where O(MeOH) represents the hydroxyl oxygen atom of methanol (Fig. 4c). In System 2, the corresponding C(PC)–O(MeOH) RDF peak remains at a similar distance but exhibits a slightly lower intensity (Fig. 4d), indicating that the presence of NaCl decreases the local density of methanol molecules around PC and weakens the ordering of the solvent structure. Furthermore, when *r* > 13.1 Å, the RDF curves of the two systems nearly overlap and the *g*(*r*) values gradually approach 1 (Fig. 4d), suggesting that the influence of NaCl on the solvent structure is primarily limited to the short-range region, with negligible effects on long-range distributions.

**Fig. 5:**
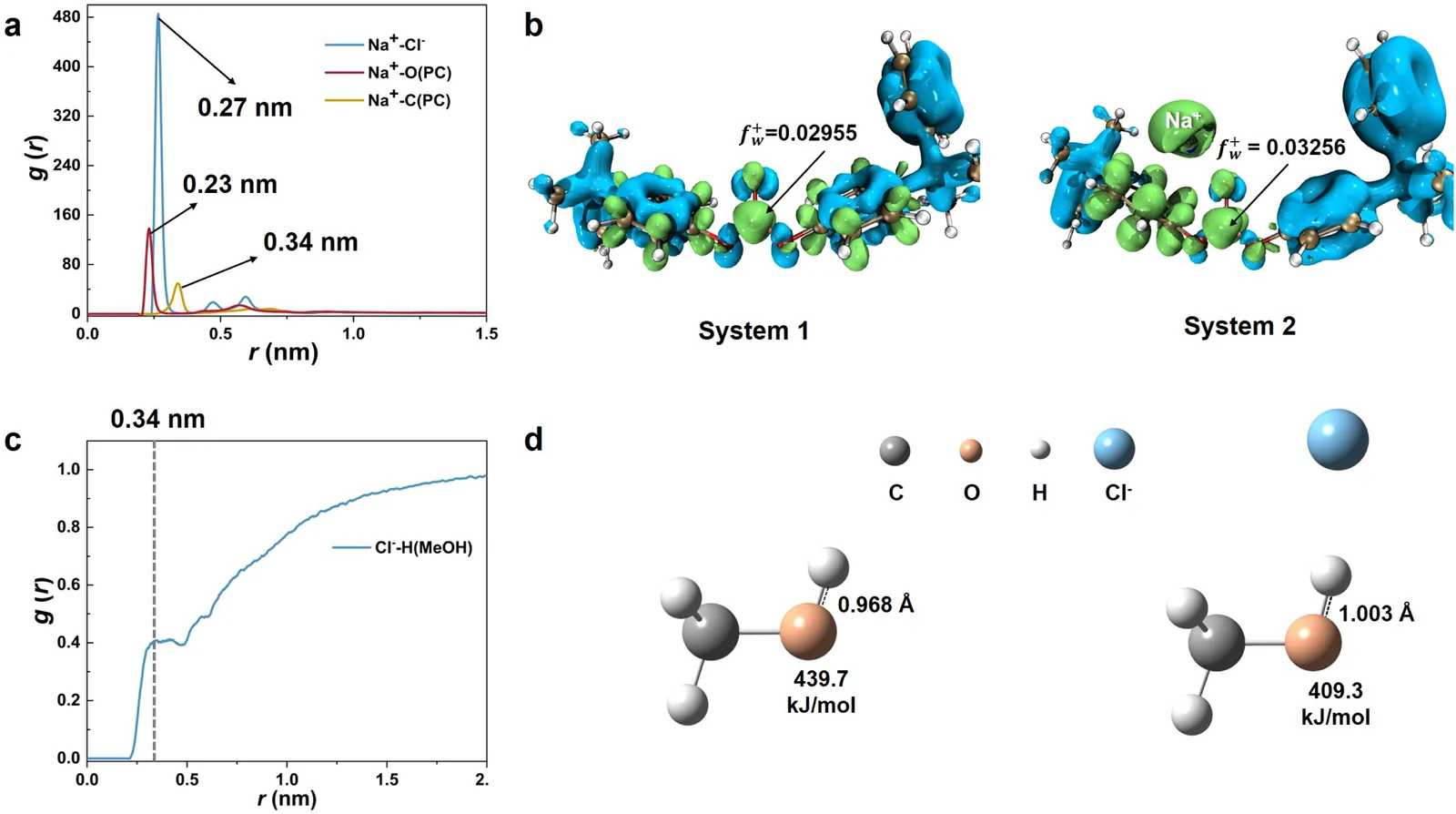
MD simulation and DFT calculation results. (a) RDFs of Na^+^ with surrounding atoms obtained from MD simulations in System 2; (b) orbital-weighted dual descriptor isosurface maps of Systems 1 and 2 (green/blue represent nucleophilic/electrophilic attack susceptibility); (c) RDFs of Cl^−^–H(MeOH) obtained from MD simulations in System 2; (d) O–H bond lengths and bond dissociation energies in Systems 1 and 2 from DFT calculation.

To further elucidate the specific roles of Na^+^ and Cl^−^ during PC methanolysis, detailed RDF analyses between these ions and the surrounding atoms were performed. The results show a distinct RDF peak at approximately 2.3 Å between Na^+^ and the carbonyl oxygen atom of PC (denoted as O(PC)) (Fig. 5a and S8), which agrees well with the typical Na–O coordination distance (2.3–2.5 Å) reported in the literature.^52,53^ In addition, the average coordination number is approximately 0.6 (Fig. S9). These results indicate that, during NaCl-promoted PC methanolysis, Na^+^ preferentially coordinates with the carbonyl oxygen of PC, thereby influencing its methanolysis behavior.^22,32,53–56^ Furthermore, as shown in Fig. 5b, orbital-weighted Fukui function and orbital-weighted dual descriptor analyses based on DFT calculations reveal that the *f*_w_^+^ value of the carbonyl carbon in PC increases significantly in the presence of Na^+^ (from 0.02955 to 0.03256). This result indicates that the introduction of Na^+^ enhances the electrophilicity of the carbonyl carbon, rendering it more susceptible to nucleophilic attack.^57–59^ Consequently, the carbonate group becomes activated, thereby facilitating cleavage of the C–O bond.

Meanwhile, Cl^−^ activate methanol primarily through interacting with the O–H bonds of methanol during PC methanolysis.^28,55,56^ As shown in Fig. 5c, a weak and broad RDF peak is observed at around 3.4 Å between Cl^−^ and the hydroxyl hydrogen atom of methanol (denoted as H(MeOH)), indicating that Cl^−^ can interact with H(MeOH). This observation suggests the possible existence of O–H⋯Cl^−^ hydrogen-bonding interactions within the system. DFT calculations reveal that, in the presence of NaCl, the O–H bond length of methanol increases from 0.968 Å to 1.003 Å, and its bond dissociation energy decreases from 439.7 kJ mol^−1^ to 409.3 kJ mol^−1^ (Fig. 5d). Meanwhile, ATR-FTIR results show a pronounced red shift of the O–H stretching vibration band (Fig. 3c). These results demonstrate that the interaction between Cl^−^ and H(MeOH) polarizes and weakens the O–H bond in methanol, thereby perturbing the intrinsic hydrogen-bonding environment of methanol and rendering it more susceptible to activation. Thus, methanol exhibits higher reactivity and an enhanced tendency to nucleophilically attack the carbonyl carbon of PC.

**Fig. 6:**
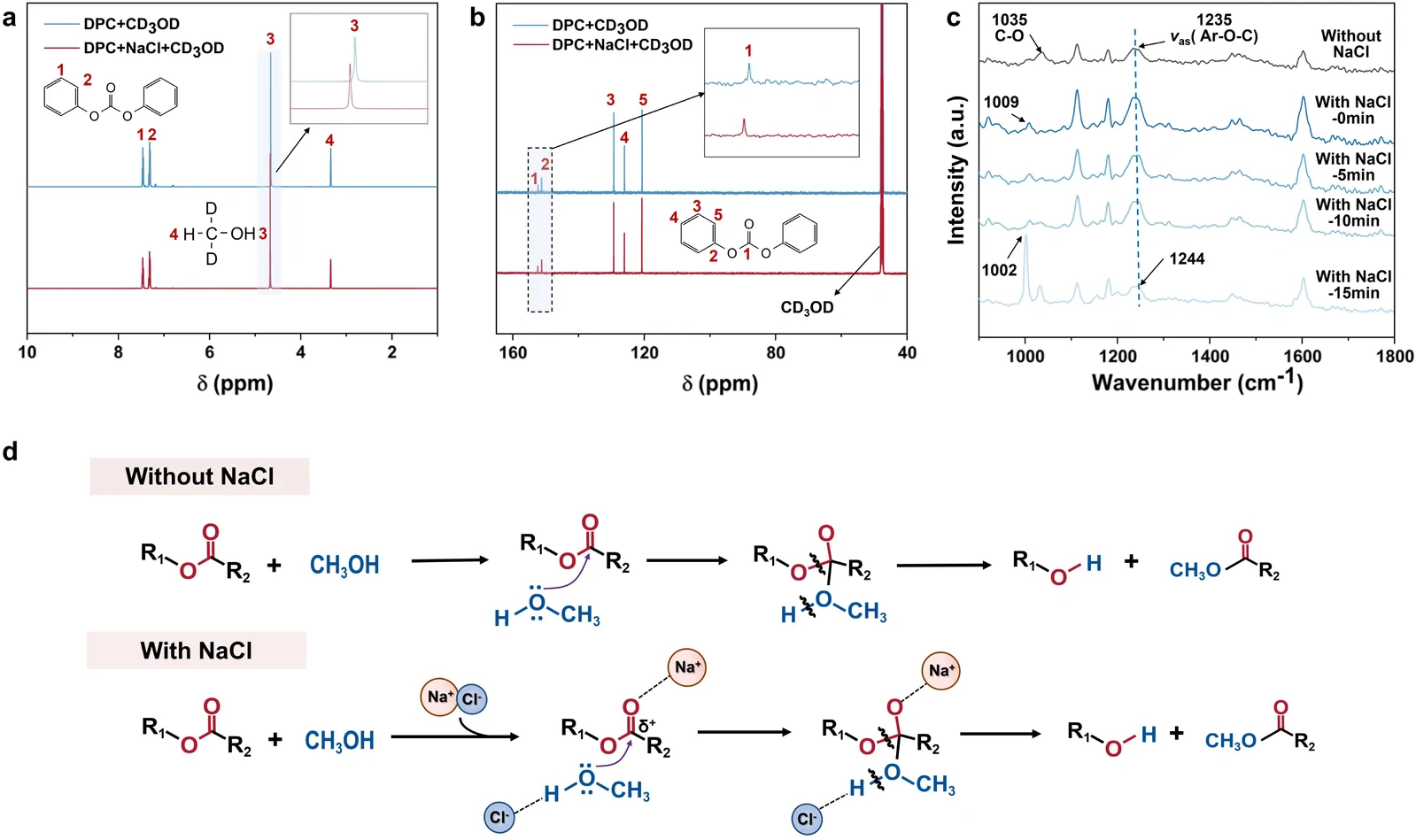
NMR and Raman spectroscopic investigation of the interactions between NaCl, methanol, and PC. (a) ^1^H NMR spectra of the interaction between methanol and NaCl, mass ratio of DPC/NaCl was 5/1 in CD_3_OD; (b) ^13^C NMR spectra of the interaction between DPC with NaCl, mass ratio of DPC/NaCl was 5/1 in CD_3_OD; (c) Raman spectra of PC methanolysis with and without NaCl. Reaction conditions: 423 K, 0.5 MPa N_2_, 1.00 g PC powder, 0.15 g NaCl, and 30.00 g methanol; (d) proposed mechanism for NaCl-promoted methanolysis.

To further validate the respective roles of Na^+^ and Cl^−^ proposed by the MD simulations and DFT calculations, additional ^1^H NMR, ^13^C NMR, and Raman spectroscopy measurements were performed. As shown in Fig. 6a, the hydroxyl proton resonance of methanol exhibits a downfield shift from 4.65 to 4.67 ppm after the addition of NaCl in the ^1^H NMR spectrum (peak 3), indicating an altered hydrogen-bonding environment that is consistent with the interaction between Cl^−^ and the hydroxyl group of methanol. Meanwhile, the carbonyl carbon resonance of diphenyl carbonate (DPC), employed as a model compound for the carbonate linkage in PC, exhibits a slight downfield shift from 152.35 to 152.40 ppm in the ^13^C NMR spectrum (Fig. 6b, peak 1), indicating a perturbed electronic environment around the carbonyl group. This observation is consistent with the interaction of Na^+^ with the carbonyl oxygen. Furthermore, Raman spectroscopy shows that, during NaCl-promoted PC methanolysis, the C–O stretching vibration of methanol (∼1035 cm^−1^)^60^ undergoes an obvious red shift to ∼1002 cm^−1^ accompanied by an increase in intensity, whereas the asymmetric Ar–O–C stretching vibration of PC (∼1235 cm^−1^)^61^ shifts to a higher wavenumber (∼1244 cm^−1^) (Fig. 6c). These changes indicate modifications in the local bonding environments of both methanol and PC, and are consistent with the respective interactions of Cl^−^ with the hydroxyl group of methanol and Na^+^ with the carbonyl oxygen of PC. Taken together with the MD simulations and DFT calculations, these results provide complementary experimental evidence for the proposed ion-pair cooperative activation mechanism, in which Cl^−^ activates the nucleophile while Na^+^ activates the substrate.

In summary, the promoting effect of NaCl on PC methanolysis originates from ion-pair cooperative mechanism (Fig. 6d): (1) Na^+^ preferentially coordinates with the carbonyl oxygen atom in the carbonate group of PC, thereby enhancing the electrophilicity of the carbonyl carbon; (2) Cl^−^ mainly interacts with the hydroxyl hydrogen of methanol through O–H⋯Cl^−^ interactions, thereby perturbing the intrinsic hydrogen-bonding network of methanol and polarizing the O–H bond. As a result, methanol becomes activated and exhibits enhanced nucleophilic attack capability toward the carbonyl carbon. It is worth noting that catalysis is achieved through the electrostatic interaction between the methanol-solvated cation and the carbonyl group, together with the hydrogen-bonding interaction between the methanol-solvated anion and methanol, which is similar to that observed in organo-salt systems.^62–64^

### Effect of different ions on methanolysis

**Fig. 7:**
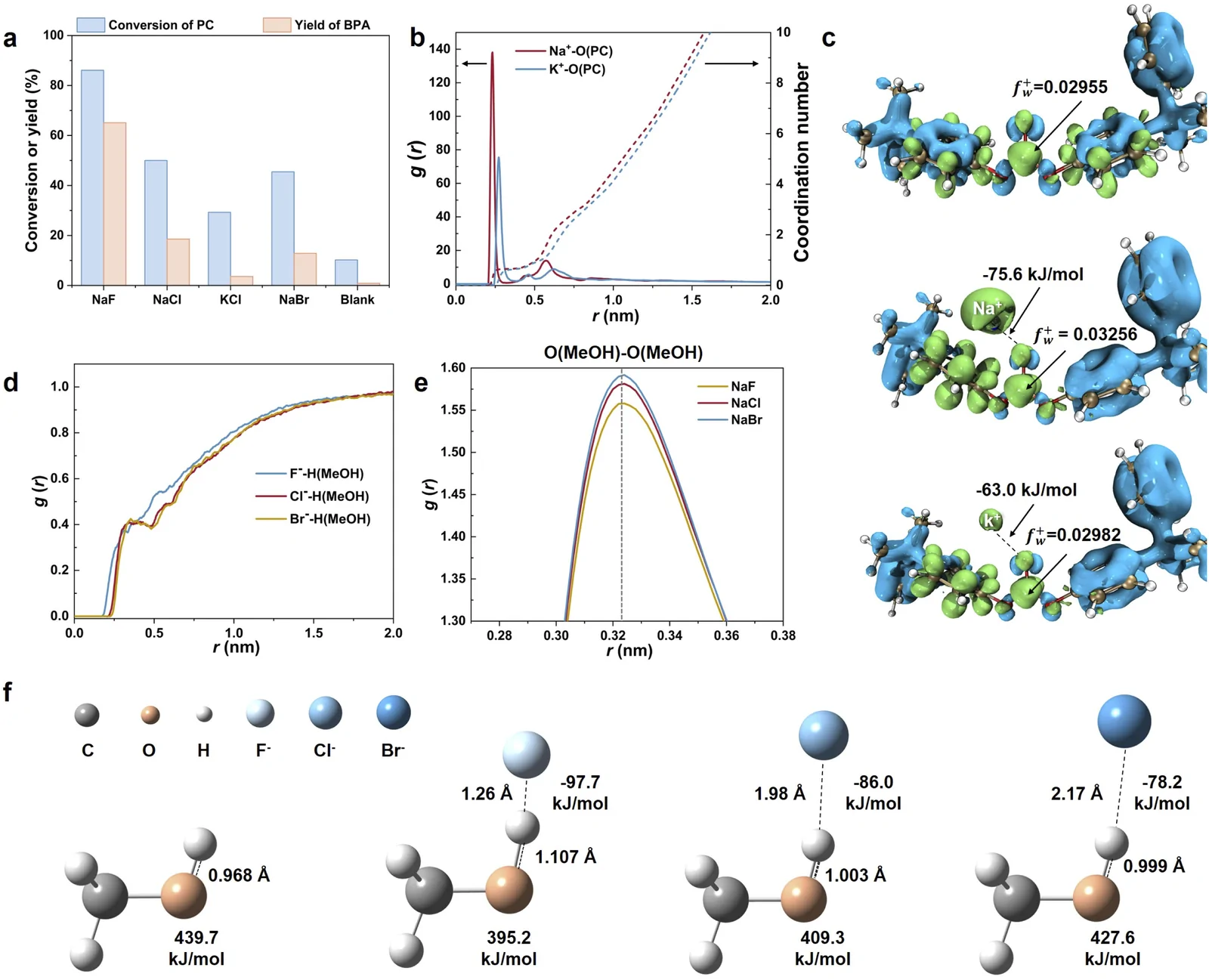
Effect of different ions on methanolysis. (a) PC methanolysis promoted by neutral sodium salts with different cations and anions. Reaction conditions: 423 K, 0.5 MPa N_2_, 15 min, 1.00 g PC powder, 2.6 mmol salts, and 30.00 g methanol; (b) RDFs and coordination number of Na^+^/K^+^–O(PC) from MD simulations; (c) orbital-weighted dual descriptor isosurfaces and interaction energies between different cations and the carbonyl oxygen from DFT calculations; (d) RDFs of different anion-H(MeOH) obtained from MD simulations; (e) RDFs of O(MeOH)–O(MeOH) under different anion systems obtained from MD simulations; (f) O–H bond lengths, bond dissociation energies, interaction distances, and interaction energies between different anions and the hydroxyl hydrogen from DFT calculations.

To investigate the effects of different ions on PC methanolysis, four representative neutral salts (NaF, NaCl, NaBr, and KCl) were selected for comparative experiments. As shown in Fig. 7a, all salts significantly promoted PC methanolysis relative to the salt-free system. Among the cations examined, NaCl exhibited higher methanolysis activity than KCl. For the anions, the activity followed the order NaF > NaCl > NaBr. These results indicate that both cationic and anionic species play crucial roles in determining the methanolysis performance of PC. To further elucidate how different cations and anions influence substrate and methanol activation, MD simulations and DFT calculations were performed.

The role of cations in PC methanolysis was investigated by analyzing the RDFs between Na^+^/K^+^ and the carbonyl oxygen atoms of PC. As shown in Fig. 7b, a distinct first coordination peak appears at approximately 2.7 Å for the K^+^–O(PC) pair, with an average coordination number of ∼0.6, indicating that K^+^, similar to Na^+^, can coordinate with the carbonyl oxygen atoms of PC. However, compared with the Na^+^–O(PC) pair, the first RDF peak of K^+^–O(PC) is less intense and shifted to a longer distance, suggesting weaker coordination between K^+^ and the carbonyl oxygen atoms. Furthermore, DFT calculations revealed that the interaction energy between the cation and the carbonyl oxygen follows the order of Na^+^ > K^+^ (Fig. 7c), indicating a stronger interaction of Na^+^ with the carbonyl oxygen than that of K^+^. This result further corroborates that K^+^ possesses a lower ability than Na^+^ to activate the carbonyl group of PC. To further evaluate the influence of K^+^ on substrate activation, orbital-weighted Fukui function and orbital-weighted dual descriptor analyses were performed (Fig. 7c). The results show that the *f*_w_^+^ value of the carbonyl carbon increases from 0.02955 in the salt-free system to 0.02982 in the presence of K^+^, indicating enhanced electrophilicity of the carbonyl carbon. Nevertheless, this value remains lower than that obtained for the Na^+^-containing system (0.03256), demonstrating that Na^+^ is more effective than K^+^ in activating the carbonyl carbon. This conclusion is consistent with the experimental observation that NaCl exhibits superior methanolysis activity compared with KCl.

The role of anions was subsequently investigated by analyzing the RDFs between F^−^, Cl^−^, and Br^−^ and the hydroxyl hydrogen of methanol. As shown in Fig. 7d, the RDF peaks gradually shift toward longer distances in the order F^−^–H(MeOH) < Cl^−^–H(MeOH) < Br^−^–H(MeOH). In addition, the peak intensity decreases in the order F^−^ > Cl^−^ > Br^−^. Furthermore, DFT calculations showed that both the interaction distances and interaction energies between the anions and the hydroxyl hydrogen of methanol exhibit the same trend as that observed in the RDF analysis (Fig. 7f), further confirming the progressively weaker interactions between the anions and methanol. These differences reflect the distinct abilities of the anions to perturb the intrinsic hydrogen-bonding network of methanol. Further evidence is provided by the RDFs of O(MeOH)–O(MeOH), which exhibit a characteristic peak at approximately 3.24 Å in all systems. Notably, the peak intensity follows the order NaBr > NaCl > NaF (Fig. 7e), suggesting that methanol molecules are most structurally ordered in the NaBr system and least ordered in the NaF system. This observation indicates that F^−^ disrupts the hydrogen-bonding network of methanol most effectively.

Consistent with the MD results, ATR-FTIR spectra show that the red shift of the O–H stretching vibration follows the order NaF > NaCl > NaBr (Fig. S10). This behavior originates from the different hydrogen-bonding interactions between the electron-rich anions and the hydroxyl proton of methanol. Acting as hydrogen-bond acceptors, F^−^, Cl^−^, and Br^−^ interact with H(MeOH) to different extents, resulting in varying degrees of O–H bond weakening. DFT calculations further reveal that the O–H bond dissociation energy decreases in the order NaBr > NaCl > NaF, whereas the O–H bond length follows the opposite trend (NaF > NaCl > NaBr) (Fig. 7f). These results demonstrate that stronger anion–methanol interactions progressively polarize and weaken the O–H bond, thereby reducing its stability and facilitating methanol activation. Consequently, the enhanced activation of methanol in the NaF system contributes to its superior methanolysis activity relative to the NaCl and NaBr systems.

In summary, the differences in methanolysis activity among the various neutral salt systems originate from their distinct abilities to activate both the PC substrate and methanol. Substrate activation is governed primarily by the strength of the coordination interaction between the cation and the carbonyl oxygen atoms of PC, which determines the electrophilicity of the carbonyl carbon. On the other hand, methanol activation depends mainly on the interaction strength between the anion and methanol, which influences the polarization and dissociation of the O–H bond. The synergistic interplay between these two activation pathways ultimately regulates the nucleophilic attack of methanol on the carbonyl carbon, leading to the different methanolysis performances observed among the neutral salt systems.

### Methanolysis promoted by natural salts

**Fig. 8:**
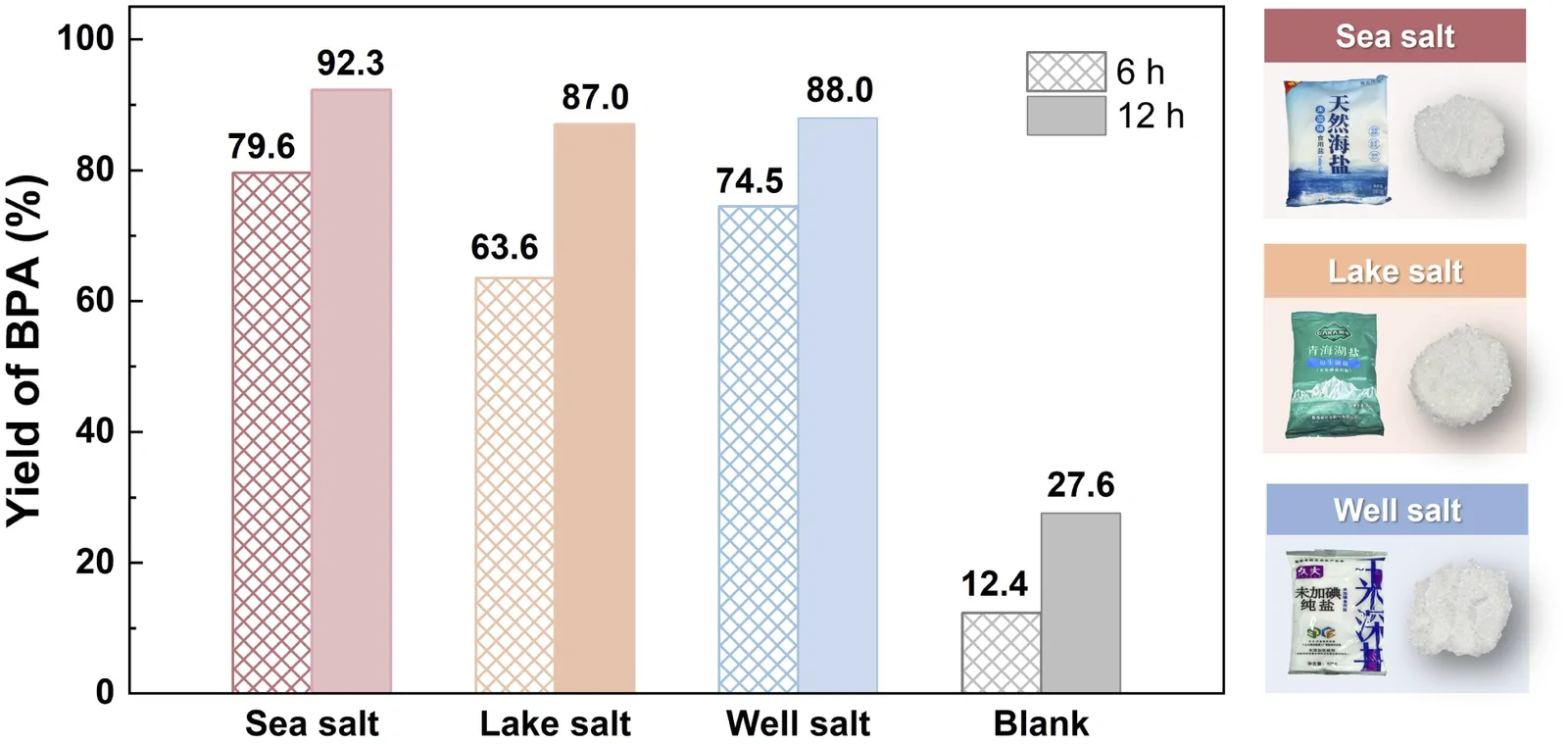
Performance of PC methanolysis promoted by natural salts. Reaction conditions: 423 K, 0.5 MPa N_2_, 1.00 g PC powder, 0.15 g salts, and 30.00 g methanol.

To evaluate the general applicability of salt-promoted PC methanolysis, several common natural salts encountered in daily life, including sea salt, lake salt, and well salt, were selected. Compared with the methanolysis of PC in the absence of additives, all three salts exhibited pronounced promoting effects on the depolymerization process. After 60 min of reaction, the BPA yields reached 79.6%, 63.6%, and 74.5% for sea salt, lake salt, and well salt, respectively. Upon extending the reaction time to 120 min, the BPA yields further increased to 92.3%, 87.0%, and 88.0% for sea salt, lake salt, and well salt, respectively (Fig. 8). These results demonstrate that natural salts can effectively promote the methanolysis of PC, highlighting the broad applicability and practical potential of salt-assisted depolymerization systems.

Furthermore, to investigate the origin of the different promoting effects of various natural salts on PC methanolysis, the ionic compositions of these salts were analyzed (Table S2). The results revealed that the superior catalytic performance of sea salt could be attributed to its higher Cl^−^ content. It is worth noting that, in addition to Na^+^ and K^+^, these natural salts also contain trace amounts of Ca^2+^ and Mg^2+^. To evaluate their potential contributions, comparative methanolysis experiments were performed using chloride salts containing different cations. The results showed that both MgCl_2_ and CaCl_2_ promoted PC methanolysis (Fig. S11), which may be attributed to the Lewis acidity of Mg^2+^ and Ca^2+^.^65^ However, because the concentrations of these cations in the natural salts are very low (typically below 0.5 wt%), their contributions to the overall catalytic performance can be regarded as negligible.

### NaCl-promoted methanolysis of real waste plastics

**Fig. 9:**
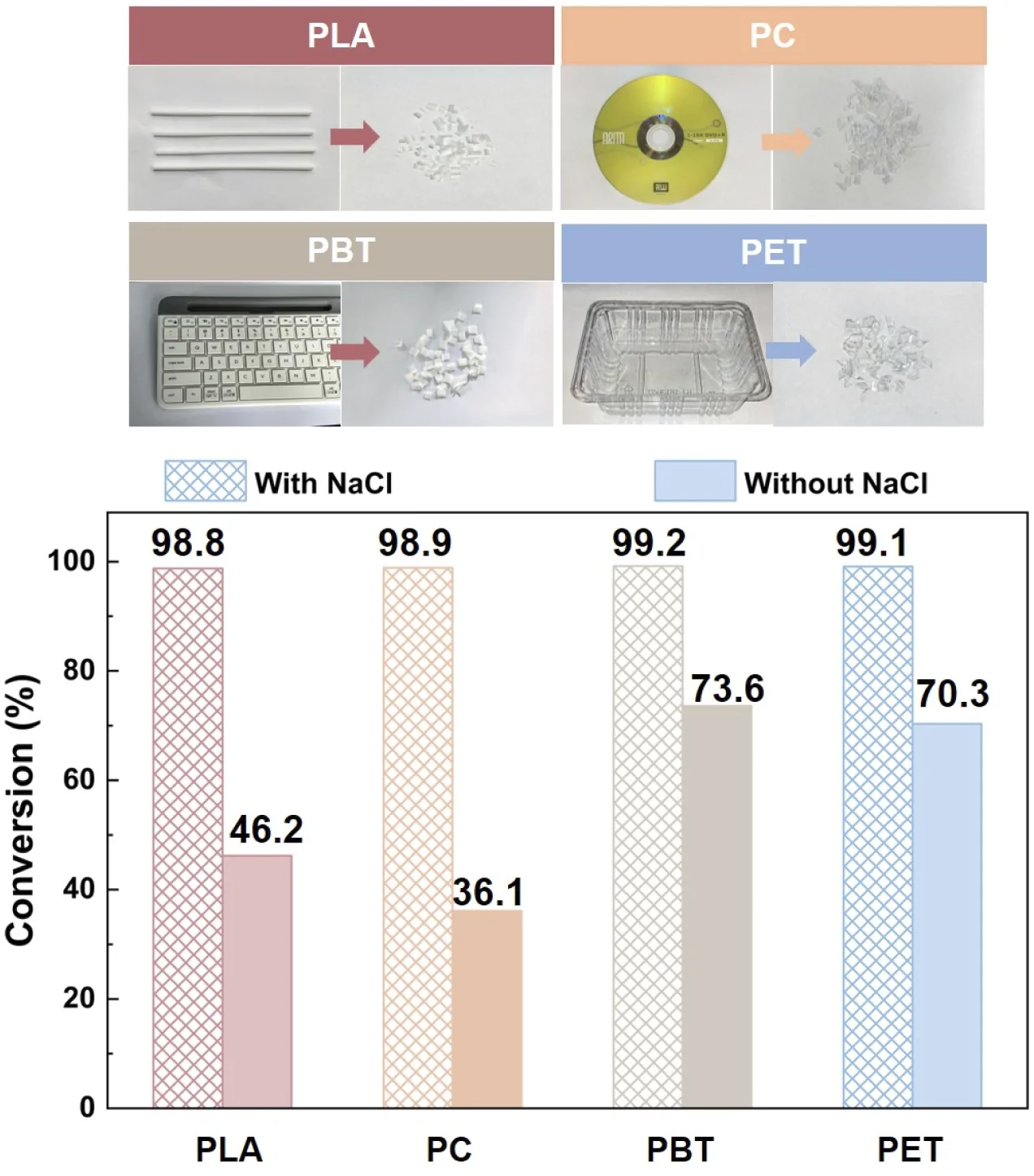
Performance of NaCl-promoted methanolysis of real waste plastics. PLA reaction conditions: 403 K, 0.5 MPa N_2_, 720 min, 1.00 g PLA fragments, 0.15 g NaCl, and 30.00 g methanol; PC reaction conditions: 423 K, 0.5 MPa N_2_, 60 min, 1.00 g PC fragments, 0.15 g NaCl, and 30.00 g methanol; PBT reaction conditions: 453 K, 0.5 MPa N_2_, 480 min, 1.00 g PBT fragments, 0.15 g NaCl, and 30.00 g methanol; PET reaction conditions: 453 K, 0.5 MPa N_2_, 480 min, 1.00 g PET fragments, 0.15 g NaCl, and 30.00 g methanol.

To evaluate the general applicability of NaCl for ester-containing plastics methanolysis, representative post-consumer plastic products, including packaging containers, DVDs, straws, and keyboard keycaps, were selected as practical sources of PET, PC, PLA, and PBT, respectively. As shown in Fig. 9, the addition of NaCl significantly enhanced the depolymerization efficiency of all four ester-containing waste plastics compared with the corresponding salt-free systems. Specifically, the conversion of PLA increased from 46.2% to 98.8% after 12 h at 130 °C. For PC, the conversion increased from 36.1% to 98.9% after 1 h at 150 °C. For PBT, the conversion improved from 73.6% to 99.2% after 8 h at 180 °C. Similarly, the conversion of PET improved from 70.3% to 99.1% after 8 h at 180 °C. Furthermore, the addition of NaCl also effectively promoted the depolymerization of mixed ester-containing plastic waste, increasing the conversion from 72.2% to 94.1% under the same reaction conditions (Fig. S12). It is worth noting that, NaCl can be readily separated and recovered through a combination of rotary evaporation and filtration (see the SI for details). Moreover, the recovered NaCl retained good catalytic activity (Fig. S13). These results demonstrate that NaCl is broadly effective in promoting the methanolysis of real-world waste ester-containing plastics, highlighting its potential as a simple, inexpensive, and versatile additive for the chemical recycling of ester-containing plastics waste streams.

## Conclusions

In summary, this work demonstrates that common neutral salts can promote the methanolysis of ester-containing plastics through an ion-pair cooperative mechanism. The main findings of this work can be summarized as follows.

(1) The ion-pair cooperative mechanism. Combined experimental investigations, *in situ* DRIFTS analyses, MD simulations, DFT calculations, nuclear magnetic resonance, and Raman spectroscopy reveal that NaCl simultaneously activates both the substrate and the nucleophile. Specifically, Na^+^ preferentially coordinates with the carbonyl oxygen atoms of the polymer, increasing the electrophilicity of the carbonyl carbon and facilitating nucleophilic attack, whereas Cl^−^ interacts with methanol through O–H⋯Cl^−^ interactions, perturbing the hydrogen-bonding network and polarizing the O–H bonds, thereby facilitating methanol activation.

(2) The structure–activity relationship. The methanolysis activity follows the order of Na^+^ > K^+^ for cations and F^−^ > Cl^−^ > Br^−^ for anions. The superior performance of Na^+^ originates from its stronger interaction with carbonyl oxygen atoms, which increases the electrophilicity of carbonyl carbon and enables more effective substrate activation. For anions, their capacity to activate methanol depends on the interaction strength with methanol, which influences the polarization and dissociation of the O–H bond.

(3) The practical applicability. The salt-promoted methanolysis strategy was extended to representative ester-containing plastics, including PC, PET, PBT, PLA. Upon NaCl addition, the conversion of PC increased from 36.1% to 98.9%, PLA from 46.2% to 98.8%, PBT from 73.6% to 99.2%, PET from 70.3% to 99.1%, demonstrating the applicability of the ion-pair cooperative mechanism for depolymerization of ester-containing plastics.

## Author contributions

Y. Huang: writing – original draft, conceptualization, investigation, data curation; J. Xie: writing – review & editing, supervision; J. Xie: writing – review & editing, funding acquisition, supervision; Y. Zhou: software, validation, visualization; Y. Lei: resources; T. Jia: visualization; J. Li: visualization; C.-A. Zhou: supervision; C. Wang: visualization; L. Song: data curation; K. Ma: visualization; H. Yue: writing – review and editing, funding acquisition; J.-J. Zou: writing – review and editing.

## Conflicts of interest

There are no conflicts to declare.

## Supplementary Material

SC-OLF-D6SC04874A-s001

## Data Availability

The data supporting this article have been included as part of the supplementary information (SI). Supplementary information is available. See DOI: https://doi.org/10.1039/d6sc04874a.
