# Supplementary material for: Ion-pair cooperativity in common neutral salts promotes methanolysis of ester-containing plastics
Source: Chem Sci. 2026 Sep 15. Online ahead of print. doi: 10.1039/d6sc04874a (PMC13591211; doi:10.1039/d6sc04874a)
Supplement: Supplementary file 1 [file SC-OLF-D6SC04874A-s001.pdf]

Supplementary Information (SI) for Chemical Science.
This journal is © The Royal Society of Chemistry 2026

Supporting Information

Ion-pair cooperativity in common neutral salts promotes methanolysis of ester-containing plastics

Yushuang Huang[a,b,c]†, Junjian Xie[d]†, Jiawei Xie[a,c,e]*, Yisong Zhou[a,c], Yuan Lei[e], Tinghao Jia[f], Jinjin Li[g], Chang-An Zhou[c], Chao Wang[c], Lei Song[c], Kui Ma[c], Hairong Yue[a,c] and Ji-Jun Zou[h]

[a] Institute of New Energy and Low-Carbon Technology, Sichuan University, Chengdu, 610207, China. ***E-mail: xiejiawei@scu.edu.cn.**

[b] College of Materials and Chemistry and Chemical Engineering, Chengdu University of Technology, Chengdu, 610059, China.

[c] School of Chemical Engineering, Sichuan University, Chengdu, 610065, China.

[d] School of Chemistry and Chemical Engineering, Northwestern Polytechnical University, Xi'an, 710072, China.

[e] State Key Laboratory of Advanced Polymer Materials (Sichuan University), Sichuan University, Chengdu, 610065, China.

[f] College of Chemical and Biological Engineering, Zhejiang University, Hangzhou, 310027, China.

[g] Research Institute of Natural Gas Technology, PetroChina Southwest Oil & Gasfield Company, Chengdu, 610299, China.

[h] School of Chemical Engineering and Technology, Tianjin University, Tianjin, 300072, China.

†These authors contributed equally to this work.

Contents

Fig. S1 HPLC chromatograms of the products detected during PC methanolysis in the presence of NaCl.

Fig. S2 HPLC chromatograms of the products detected during PET methanolysis in the presence of NaCl.

Fig. S3 GC chromatograms of the products detected during PLA methanolysis in the presence of NaCl.

Fig. S4 HPLC chromatograms of the products detected during PBT methanolysis in the presence of NaCl.

Fig. S5 GC-MS spectra of products detected in PC methanolysis.

Fig. S6 Mass spectrum of the dimethyl carbonate (DMC) obtained from PC methanolysis.

Fig. S7 Mass spectrum of the bisphenol A (BPA) obtained from PC methanolysis.

Fig. S8 RDFs of Na^+ with surrounding atoms obtained from MD simulations in System 2.

Fig. S9 Average coordination number of Na^+ with surrounding atoms obtained from MD simulations in System 2.

Fig. S10 ATR-FTIR spectra of methanol with different types of salts.

Fig. S11 PC methanolysis promoted by chloride salts with different cations.

Fig. S12 Performance of NaCl-promoted methanolysis of mixed ester-containing plastic waste.

Fig. S13 Recyclability of recovered NaCl in PC methanolysis.

Table S1. GPC results of PC powder before and after methanolysis.

Table S2. Ion concentrations in various salt solutions.

Table S3. Orbital-weighted Fukui functions and orbital-weighted dual descriptors of each atom in the PC molecule in System 1.

Table S4. Orbital-weighted Fukui functions and orbital-weighted dual descriptors of Na^+ and each atom in the PC molecule in System 2.

Table S5. Orbital-weighted Fukui functions and orbital-weighted dual descriptors of K^+ and each atom in the PC molecule.

Methods

Materials and chemical reagents. PC powder was purchased from Dongguan Ruixiang Plastic Co., Ltd. Sodium chloride (NaCl), potassium chloride (KCl), anhydrous magnesium chloride ($MgCl_2$) and anhydrous calcium chloride ($CaCl_2$) were bought from Chengdu Cologne Chemicals Co., Ltd. Methanol, ethanol and sodium bromide (NaBr) were purchased from Shanghai Titan Scientific Co., Ltd. Sodium fluoride (NaF) and diphenyl carbonate (DPC) were obtained from Shanghai Adamas Reagent Co., Ltd. The natural salts used in this study consist of sea salt, lake salt and well salt. Specifically, sea salt was purchased from Shandong Zhenghao Salt Industry Technology Co., Ltd., lake salt from Anhui Banma Ecological Agriculture Technology Co., Ltd., and well salt from Zigong Yandu E-commerce Co., Ltd. Pure water is prepared in-house by the laboratory.

Salt-promoted methanolysis of ester-containing plastics. The methanolysis of ester-containing plastics (PC powder and real plastic products of PET, PC, PLA and PBT) was carried out in a 50 mL high-pressure stainless-steel autoclave. Typically, ester-containing plastics, salts (NaCl, KCl, NaF, NaBr, $CaCl_2$, $MgCl_2$, and various natural salts) and methanol were charged into the reactor. The mass ratio of ester-containing plastics to methanol was fixed at m(PC):m(CH_3OH) = 1:30, and 2.6 mmol of salt was added. The reactor was purged three times with N_2 to remove residual air, followed by pressurization with N_2 to 0.5 MPa. The reaction was conducted at a specified temperature with continuous stirring at 400 rpm (PLA: 403 K, PC:423 K, PBT and PET: 453K). When the reaction was completed, the autoclave was cooled in a water bath to room temperature, after which the liquid products and the residual solids were recovered. The liquid products were analyzed by gas chromatography (GC), gas chromatography-mass spectrometry (GC-MS), or high-performance liquid chromatograph (HPLC). The residual solids were analyzed by gel permeation chromatography (GPC) and scanning electron microscopy (SEM). The investigations of reaction time and NaCl concentration for PC methanolysis were

conducted following the same procedure. In addition, when processing real ester-containing plastics, the materials were first cut into small pieces, then washed sequentially three times with ethanol and deionized water, and finally dried prior to use in the methanolysis experiments.

Recovery of NaCl. After completion of the methanolysis reaction, the reaction mixture was collected and subjected to rotary evaporation at 45 °C to remove excess methanol. As methanol was gradually removed, the dissolved NaCl progressively crystallized and precipitated from the reaction mixture. Deionized water was then added to dissolve and remove the precipitated NaCl, followed by filtration to separate the solid depolymerization products from the NaCl-containing aqueous phase. Finally, the aqueous filtrate was evaporated under ambient conditions to recover NaCl as crystal. It is worth noting that, for the PLA methanolysis system, the methanolysis product methyl lactate (ML) is soluble in water and remains in the liquid state at room temperature. Therefore, NaCl in this system could be readily separated simply by filtering the residue obtained after rotary evaporation.

GC analysis. The liquid-phase products from PLA methanolysis were analyzed using a gas chromatograph (GC-2010 Pro, Shimadzu) equipped with an SH-5Sil capillary column (30 m × 0.25 mm × 0.25 μm) and a flame ionization detector (FID). The initial oven temperature was set at 50 °C and ramped to 280 °C at a heating rate of 10 °C/min, followed by a 5 min hold. The methanolysis product, methyl lactate (ML), was quantified using an external standard method.

GC-MS analysis. To further qualitatively identify the reaction products, gas chromatography-mass spectrometry (GC-MS, Shimadzu GCMS-QP2020) equipped with an Rtx-5MS capillary column (50 m × 0.25 mm × 0.25 μm) was employed to analyze the product composition based on their molecular ion peaks and mass spectra.

HPLC analysis. After completion of the methanolysis of PC, PET, and PBT, the reaction mixture was filtered to collect the liquid-phase products. An aliquot of the filtrate was then

diluted with methanol to an appropriate concentration for analysis. Notably, to ensure complete dissolution of the methanolysis products (BPA and DMT) in the methanol solvent, an excess amount of methanol was first added to fully dissolve the products prior to dilution. Quantitative analysis was carried out using an EazySep-3030 HPLC equipped with a GS-120-5-C18-AP column and a UV-vis detector. For BPA analysis, a mixture of HPLC-grade methanol and water (A volume ratio of 8:2) was used as the mobile phase. The column temperature was maintained at 40 °C, the flow rate was set to 1 mL/min, and detection was performed at the maximum absorption wavelength (λ = 270 nm). For DMT analysis, HPLC-grade methanol and water (A volume ratio of 7:3) were used as the mobile phase under otherwise identical conditions, with detection at λ = 254 nm. Quantification of the target compounds was performed using external calibration curves constructed from standard solutions of known concentrations.

GPC analysis. The molecular weight distributions of the pristine PC and the resulting oligomers were analyzed by GPC (Agilent 1260). Prior to analysis, both PC and its oligomers were dissolved in dichloromethane.

In situ DRIFTS experiment. To investigate the role of NaCl in PC methanolysis, in situ DRIFTS was performed. For sample preparation, PC powder was used as the solid sample in System 1, while System 2 consisted of a mixture of PC powder and NaCl with a mass ratio of 1:1. In situ DRIFTS measurements were carried out on a Bruker Tensor 27 spectrometer. Prior to measurement, the samples were pretreated by purging with N_2 at room temperature for 30 min to remove trace amounts of adsorbed moisture and other impurities from the surface. After the spectra stabilized, methanol vapor was continuously introduced by passing the inlet gas stream through a bubbling bottle containing liquid methanol, and the system was maintained at room temperature for 40 min to ensure saturation adsorption of methanol. A background spectrum was then collected once the signal became stable. During the measurement, methanol vapor was continuously

introduced into the in situ cell. The temperature was increased from room temperature to 150 °C at a heating rate of 5 °C/min, followed by isothermal holding at 150 °C for 1 h. Spectra were collected every 5 min during both the heating and isothermal stages over a range of 4000-400 cm^{-1}, and each spectrum represents the average of 64 scans.

ATR-FTIR spectroscopy. To further investigate the effect of different salts (NaCl, NaF, and NaBr) on methanol, FTIR spectroscopy was performed. The measurements were carried out on a Nicolet iS50 spectrometer over a scanning range of 4000-400 cm^{-1}, and all spectra are presented as the average of 64 scans.

MD simulations. First, a PC chain model with an appropriate degree of polymerization of 3 was constructed. Subsequently, the structures of the PC chain and methanol were optimized using ORCA [1], and the RESP charges of the polymer and methanol were fitted using Multiwfn [2]. The parameters for molecular simulations were generated based on the GAFF2 force field [3], while the parameters for all ions were adopted from the literature [4]. The simulation box was constructed using the GROMACS package [5], with dimensions of $6 \times 6 \times 6$ nm^3 and periodic boundary conditions applied in all three spatial directions. Each system consisted of 20 PC chains, 50 cations, 50 anions, and 3493 methanol molecules. After construction of the simulation box, all MD simulations were performed using the GROMACS 2020.3 software package. Following energy minimization, a 60 ns MD simulation was carried out in the NVT ensemble with a time step of 2 fs. During the simulations, the temperature was maintained at 298.15 K using the V-rescale thermostat, while the pressure was controlled using the Berendsen method. Electrostatic interactions were treated using the particle mesh Ewald (PME) method, and the cutoff radius for long-range van der Waals interactions was set to 12 Å. After completion of the simulations, further analyses were conducted to obtain the radial distribution functions (RDFs), and trajectory snapshots were visualized using VMD [6].

DFT calculations. Quantum chemical calculations were performed using the Gaussian

software package to investigate the synergistic mechanism of substrate activation and methanol activation by different ions during PC methanolysis [7]. All calculations were performed using the B3LYP method (Becke, three-parameter, Lee-Yang-Parr), a hybrid generalized gradient approximation functional [8-10]. The def2-SVP basis set was employed for geometry optimization and vibrational frequency analysis, while the def2-TZVP basis set was used for single-point energy calculations [11,12]. For the Na^+- and K^+-containing systems, the interaction energies between each cation and the carbonyl oxygen atom of PC were calculated. Furthermore, the orbital-weighted Fukui functions and orbital-weighted dual descriptors of each system were calculated using the Multiwfn program. For systems containing halide anions, the minimally augmented ma-TZVP basis set with diffuse functions was applied [11,13]. Thermodynamic parameters were evaluated using the SMD implicit solvation model with methanol as the solvent. Based on the optimized structures, the interaction distances and interaction energies between the halide anions and the hydroxyl hydrogen of methanol were further analyzed. In addition, the methanol O-H bond lengths and the corresponding O-H bond dissociation energies (BDEs) in different systems were calculated to evaluate the influence of anion interactions on O-H bond stability.

NMR spectra. ^1H NMR and ^{13}C NMR test were recorded on a 600MHz Bruker spectrometer using deuterated methanol (CD_3OD) as solvent.

Raman spectroscopy. Raman spectra of the samples before and after PC methanolysis were recorded using a Renishaw inVia Raman spectrometer equipped with a 785 nm laser. The spectra were collected over the range of 4000-100 cm^{-1} with an integration time of 20 s, a laser power of 1 mW, and one accumulation.

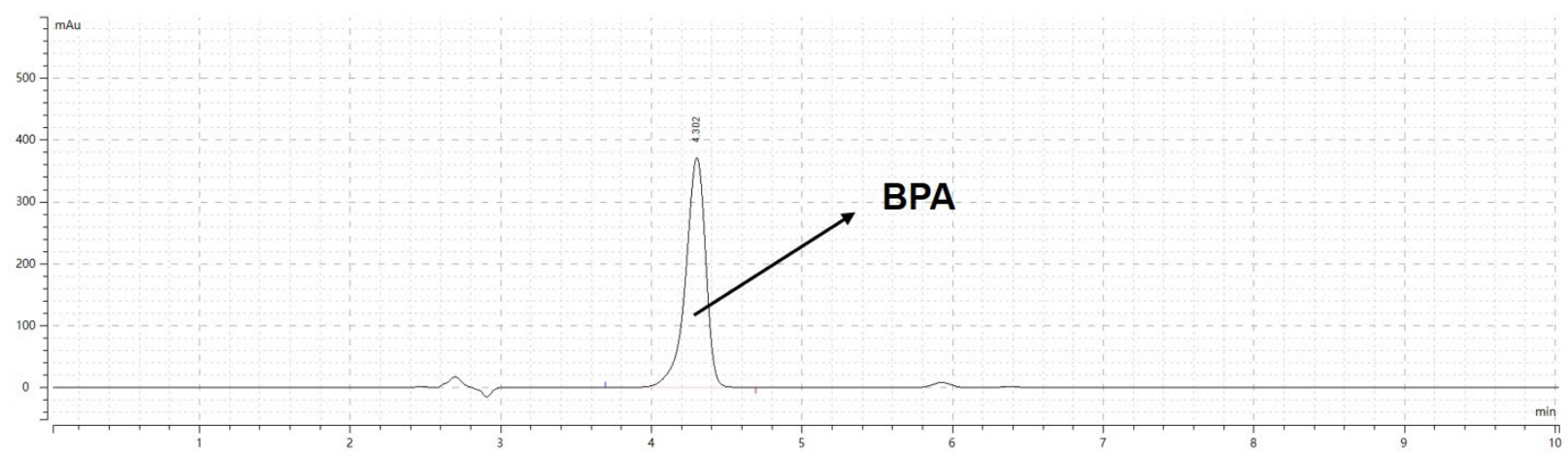

Fig. S1 HPLC chromatograms of the products detected during PC methanolysis in the presence of NaCl. Reaction conditions: 423 K, 0.5 MPa N_2, 1 h, 1.00 g PC fragments, 0.15 g NaCl, and 30.00 g methanol.

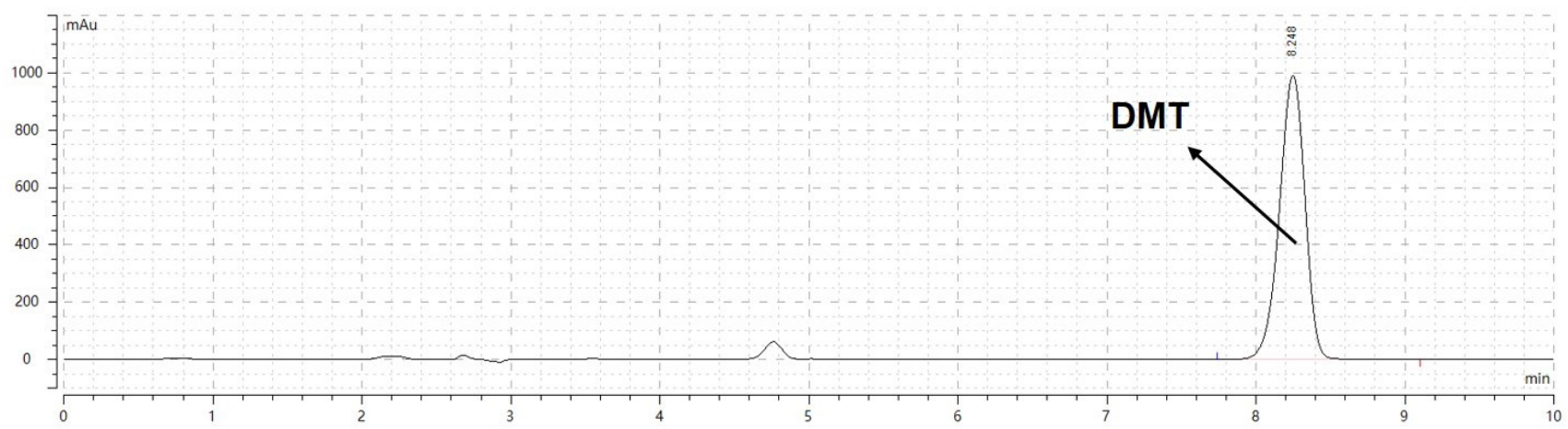

Fig. S2 HPLC chromatograms of the products detected during PET methanolysis in the presence of NaCl. Reaction conditions: 453 K, 0.5 MPa N_2, 8 h, 1.00 g PET fragments, 0.15 g NaCl, and 30.00 g methanol.

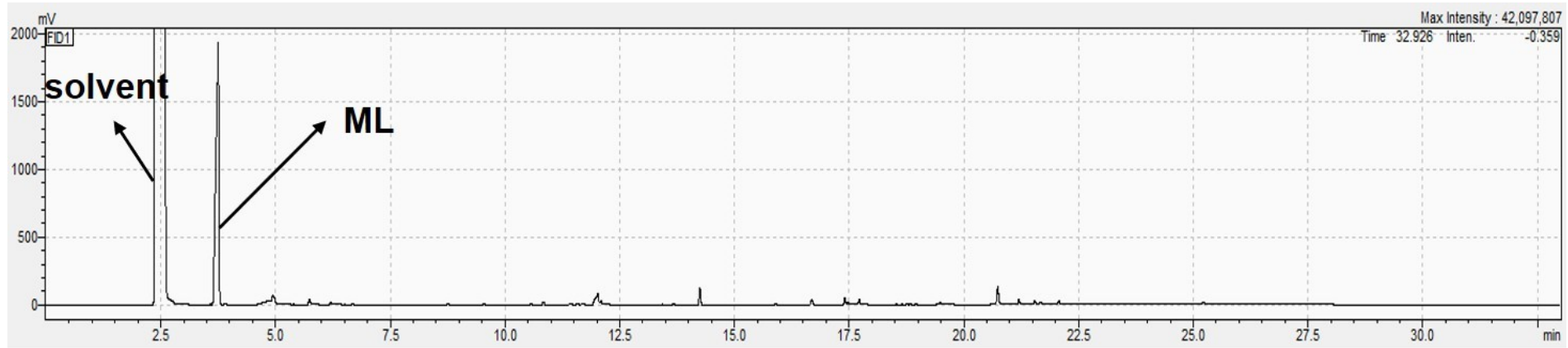

Fig. S3 GC chromatograms of the products detected during PLA methanolysis in the presence of NaCl. Reaction conditions: 403 K, 0.5 MPa N_2, 12 h, 1.00 g PLA fragments, 0.15 g NaCl, and 30.00 g methanol.

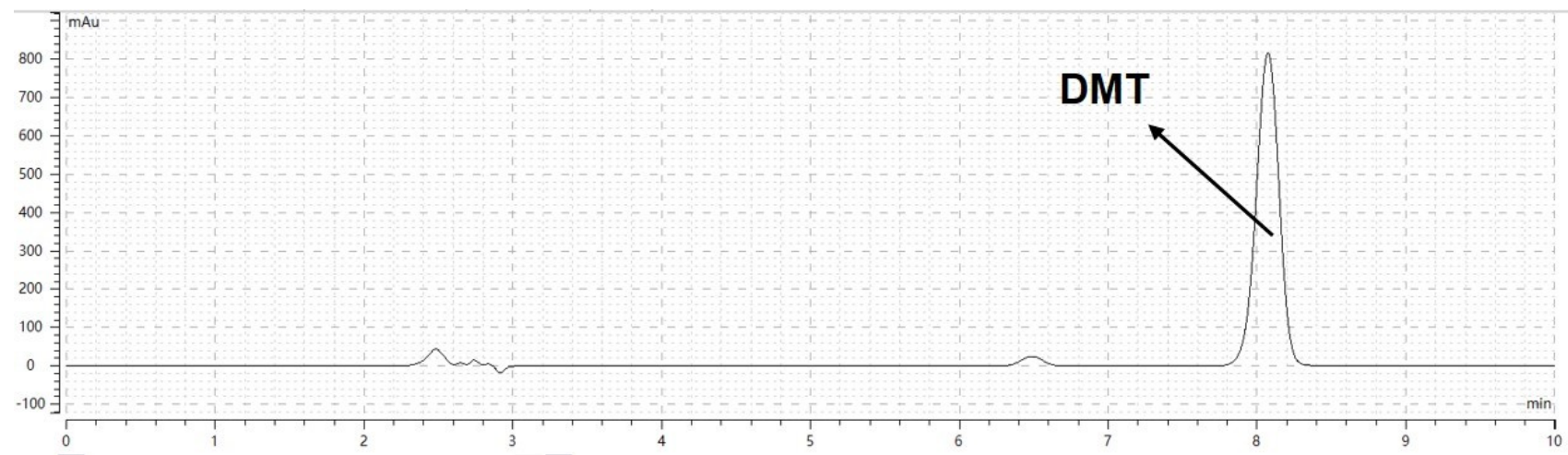

Fig. S4 HPLC chromatograms of the products detected during PBT methanolysis in the presence of NaCl. Reaction conditions: 453 K, 0.5 MPa N_2, 8 h, 1.00 g PBT fragments, 0.15 g NaCl, and 30.00 g methanol.

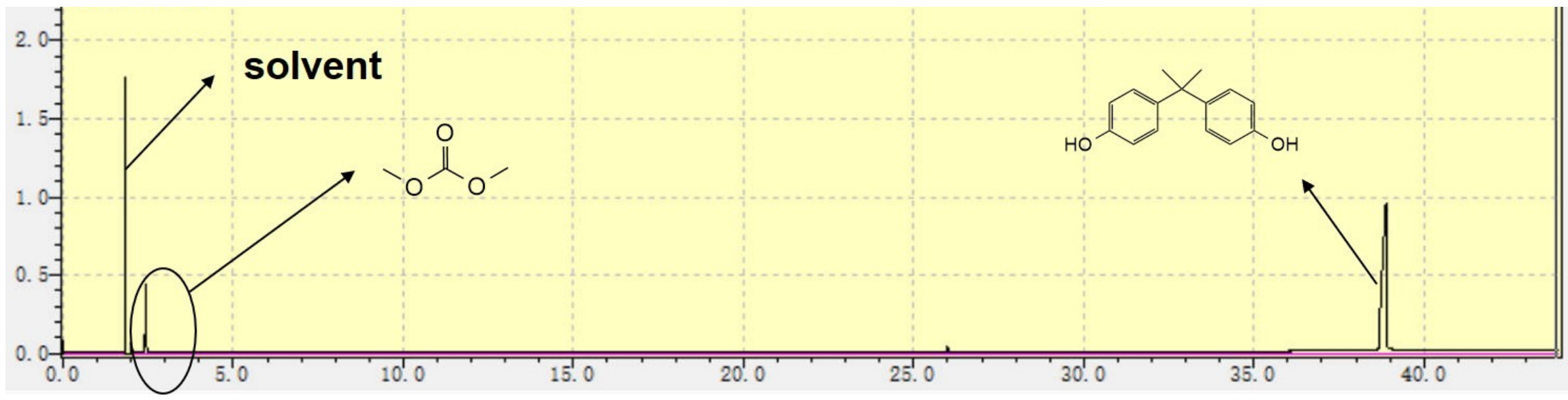

Fig. S5 GC-MS spectra of products detected in PC methanolysis. Reaction conditions: 423 K, 0.5 MPa N_2, 1 h, 1.00 g PC fragments, 0.15 g NaCl, and 30.00 g methanol.

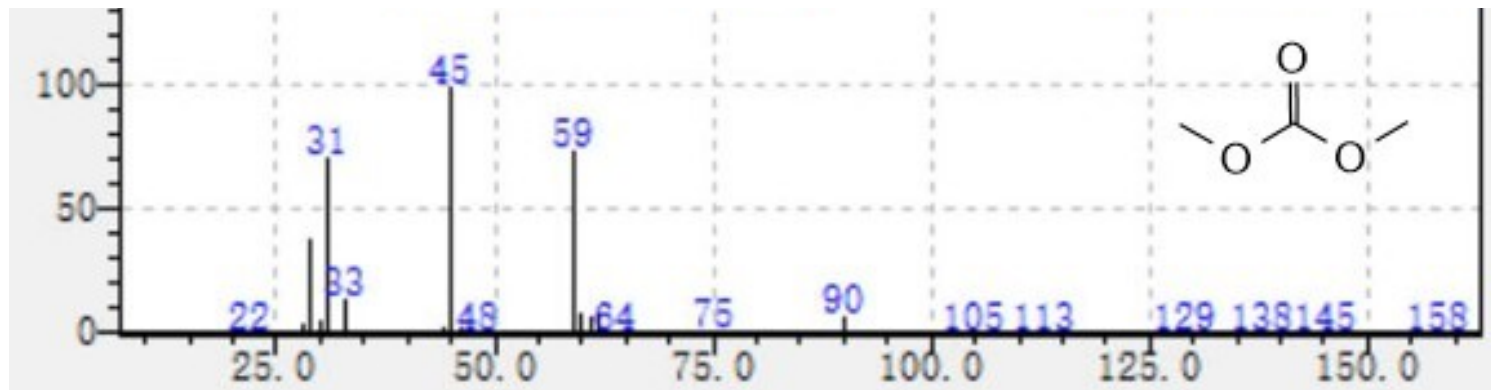

Fig. S6 Mass spectrum of the dimethyl carbonate (DMC) obtained from PC methanolysis. Reaction conditions: 423 K, 0.5 MPa N_2, 1 h, 1.00 g PC fragments, 0.15 g NaCl, and 30.00 g methanol.

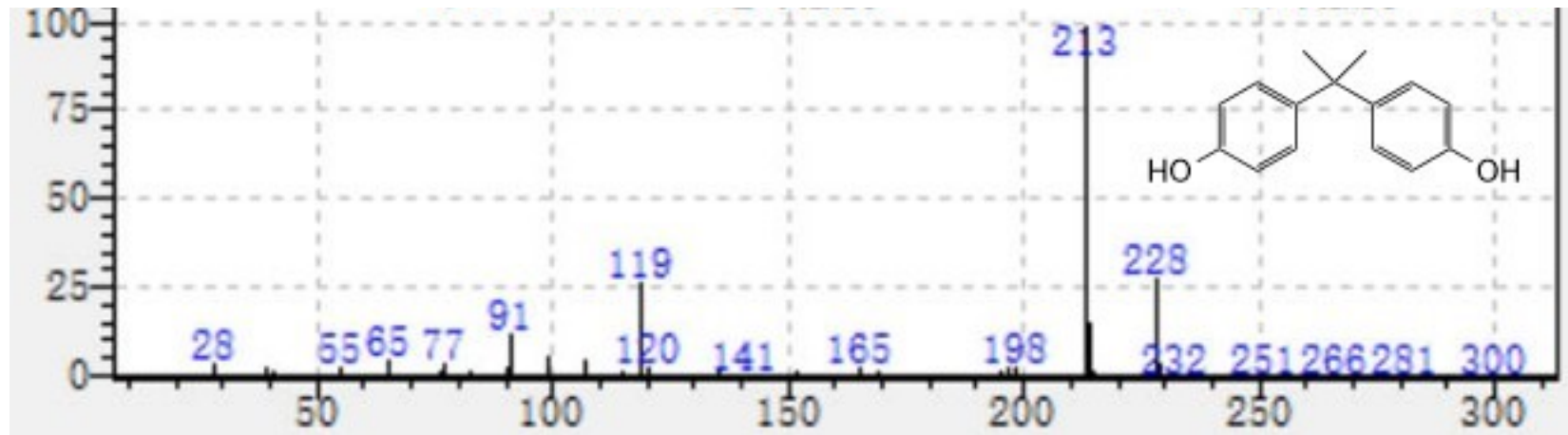

Fig. S7 Mass spectrum of the bisphenol A (BPA) obtained from PC methanolysis. Reaction conditions: 423 K, 0.5 MPa N_2, 1 h, 1.00 g PC fragments, 0.15 g NaCl, and 30.00 g methanol.

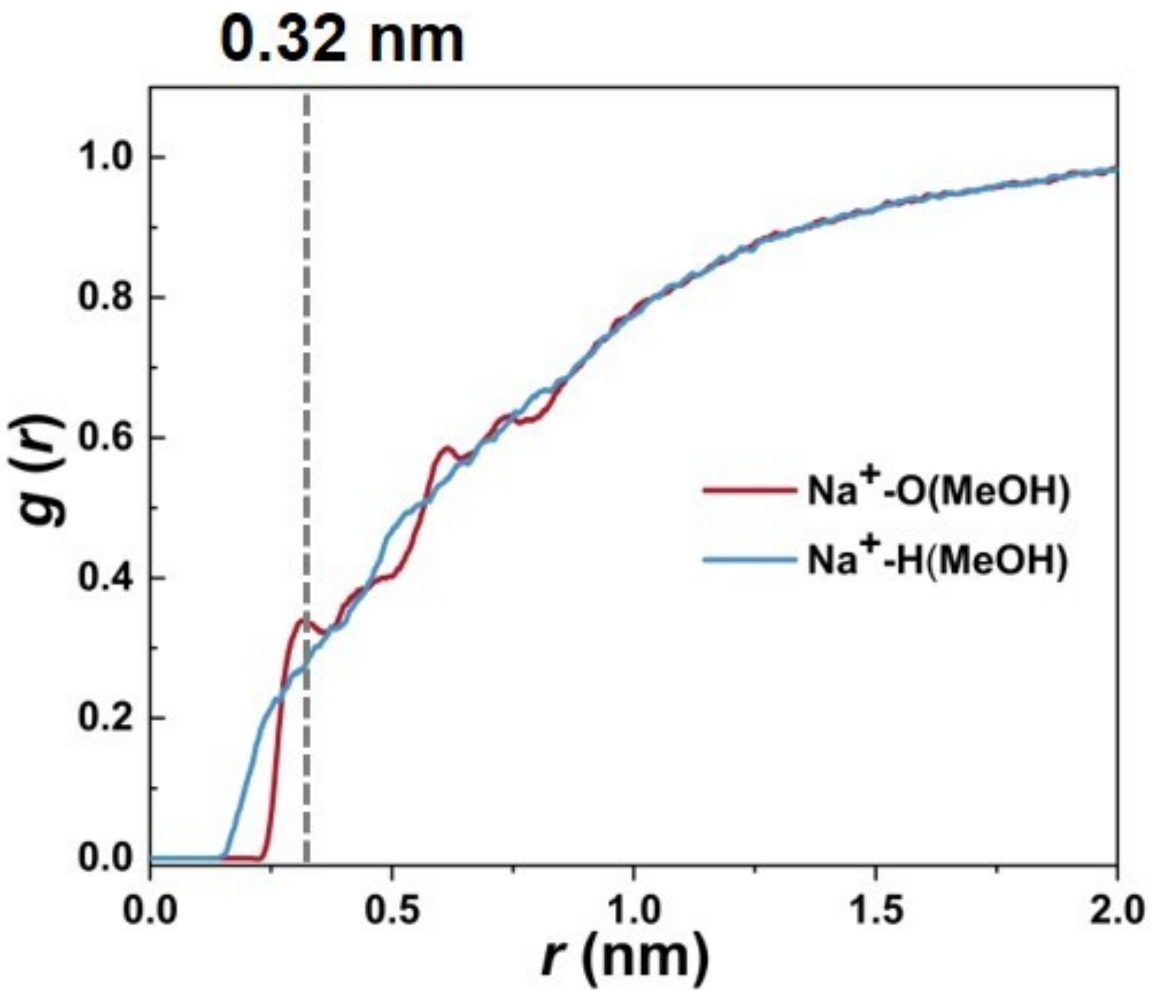

Fig. S8 RDFs of Na^{+} with surrounding atoms obtained from MD simulations in System 2.

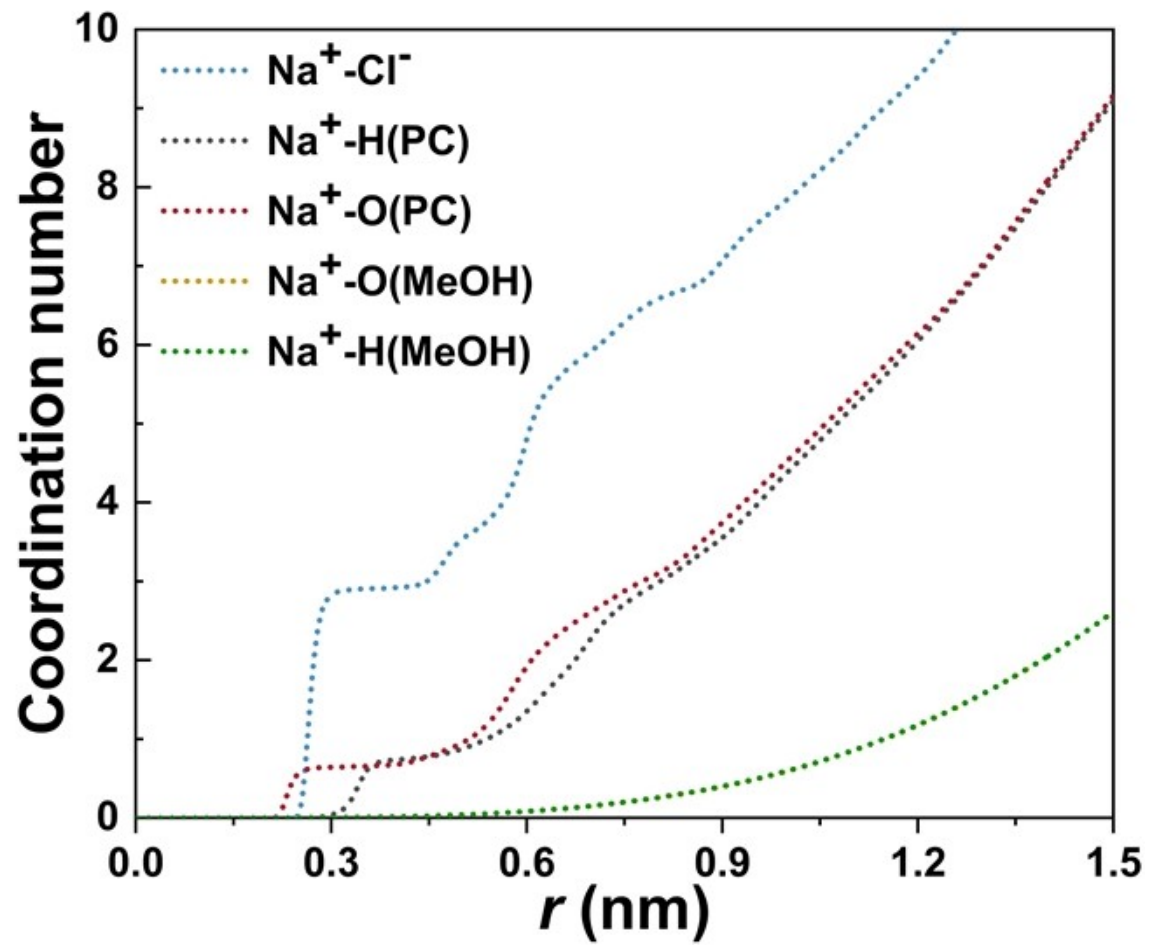

Fig. S9 Average coordination number of Na^+ with surrounding atoms obtained from MD simulations in System 2.

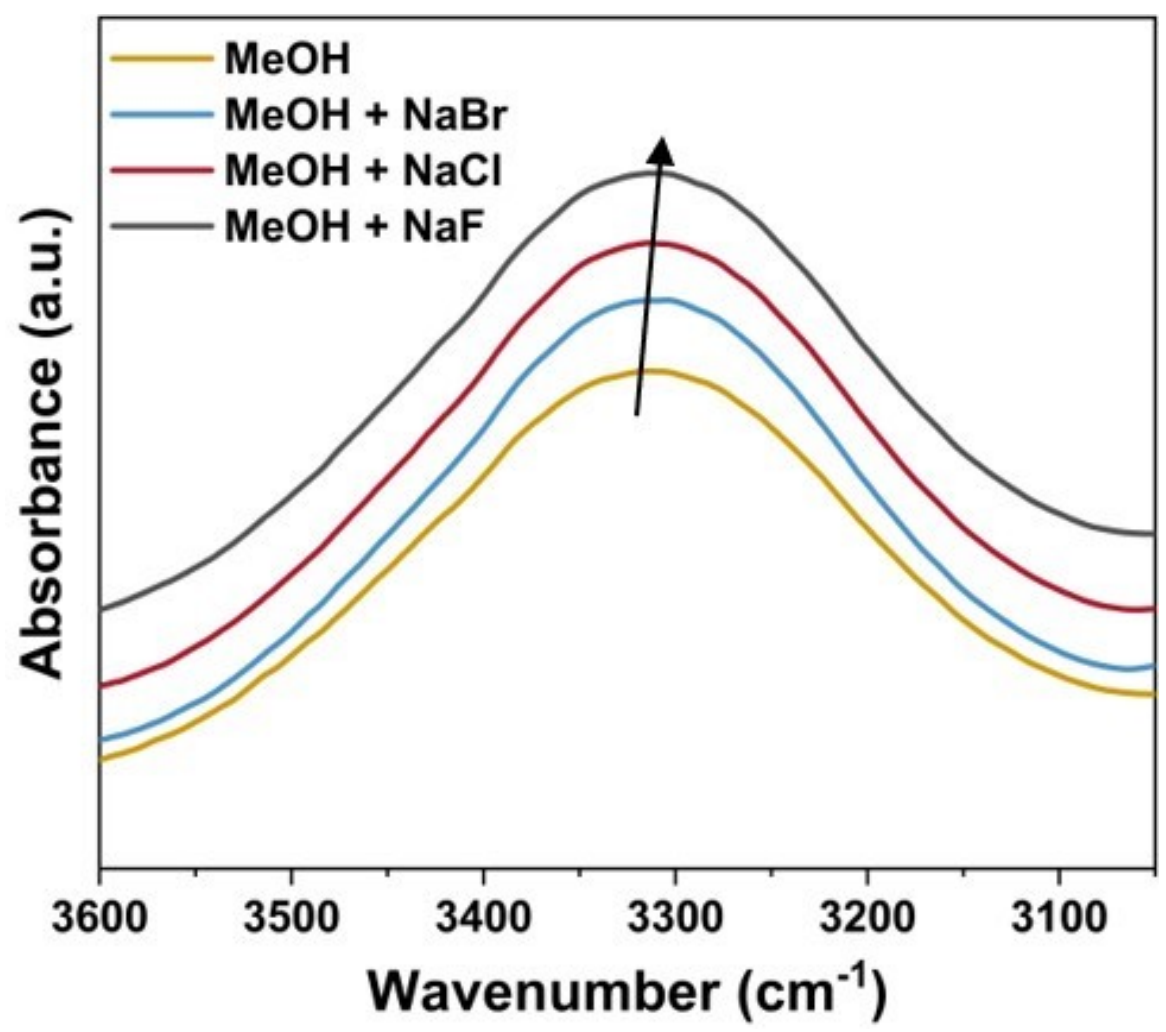

Fig. S10 ATR-FTIR spectra of methanol with different types of salts.

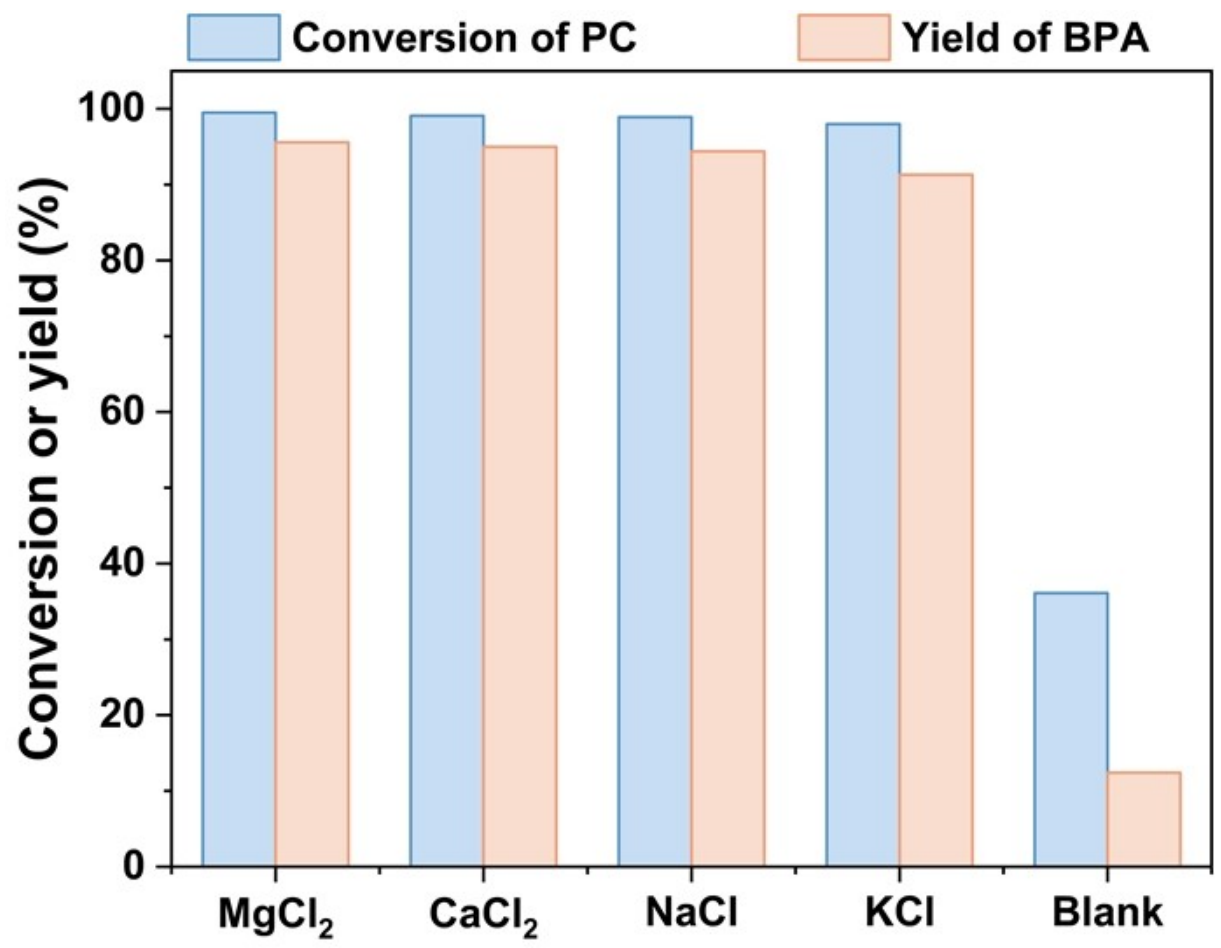

Fig. S11 PC methanolysis promoted by chloride salts with different cations. Reaction conditions: 423 K, 0.5 MPa N_2, 60 min, 1.00 g PC powder, and 30.00 g methanol. The amounts of the salts were calculated based on a fixed chloride ion content of 2.6 mmol.

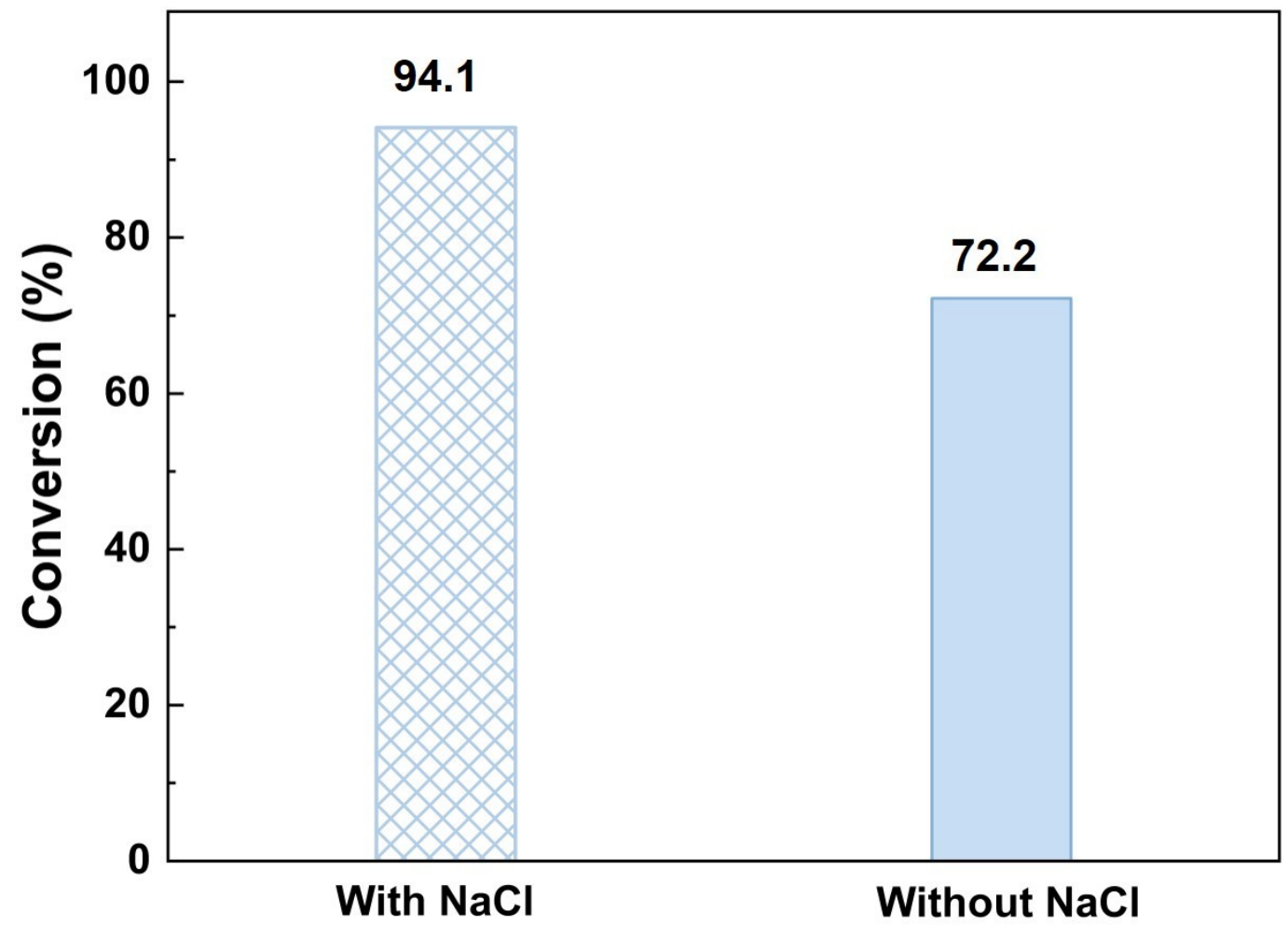

Fig. S12 Performance of NaCl-promoted methanolysis of mixed ester-containing plastic waste. Reaction conditions: 453 K, 0.5 MPa N_2, 720 min, the mass ratio of PLA, PC, PBT, PET fragments, and methanol was 1:1:1:1:120.

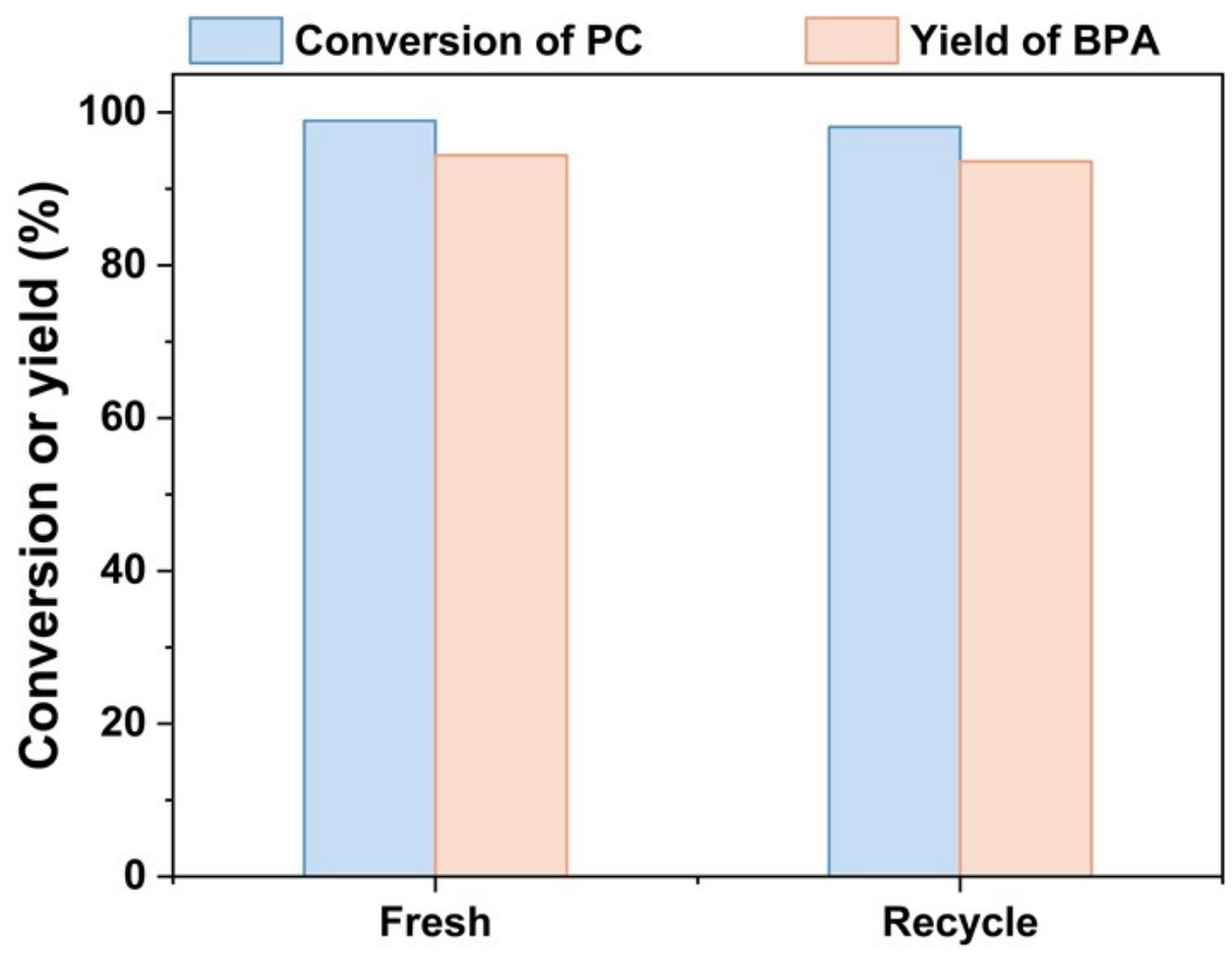

Fig. S13 Recyclability of recovered NaCl in PC methanolysis. Reaction conditions: 423 K, 0.5 MPa N_2, 60 min, 1.00 g PC powder, 0.15 g NaCl, and 30.00 g methanol.

Table S1. GPC results of PC powder before and after methanolysis.

Sample name	M_w / Da	M_n / Da	PDI
PC powder	35976	15226	2.363
PC-10 min	10593	4117	2.573
PC-15 min	9313	3500	2.661

Table S2. Ion concentrations in various salt solutions.

Sample name	Ion concentrations / mg/L						
	Ca^{2+}	Mg^{2+}	K^{+}	Na^{+}	SO_4^{2-}	F^{-}	Cl^{-}
Sea salt	2.005	0.404	0.504	341.231	2.488	0.009	626.570
Lake salt	0.726	0.582	0.550	382.692	0.662	0.009	571.757
Well salt	1.789	0.218	0.611	413.094	0.997	0.034	619.458

Measured by ion chromatography (ICS-900, Dionex Aquion).

Table S3. Orbital-weighted Fukui functions and orbital-weighted dual descriptors of each atom in the PC molecule in System 1.

Atom index	f_w^-	f_w^+	Δf_w
1(C)	0.03686	0.02884	-0.00802
2(C)	0.03491	0.02987	-0.00504
3(C)	0.03616	0.02816	-0.00800
4(C)	0.03766	0.02817	-0.0095
5(C)	0.03782	0.02993	-0.00789
6(C)	0.03386	0.02906	-0.0048
7(C)	0.01264	0.0072	-0.00544
8(C)	0.03551	0.0304	-0.00512
9(C)	0.0082	0.00555	-0.00265
10(C)	0.0082	0.00545	-0.00275
11(C)	0.0313	0.03224	0.00094
12(C)	0.0307	0.03354	0.00283
13(C)	0.03149	0.03073	-0.00075
14(C)	0.02973	0.03312	0.00339
15(C)	0.03239	0.03391	0.00152
16(O)	0.01304	0.01174	-0.0013
17(C)	0.00566	0.02955	0.0239
18(O)	0.01287	0.01179	-0.00107
19(O)	0.01457	0.01697	0.0024
20(C)	0.03138	0.03079	-0.00058
21(C)	0.03057	0.03362	0.00305
22(C)	0.03123	0.03231	0.00108
23(C)	0.03536	0.03047	-0.0049

24(C)	0.03231	0.03399	0.00168
25(C)	0.02961	0.03319	0.00358
26(C)	0.01263	0.00721	-0.00543
27(C)	0.03778	0.02809	-0.00969
28(C)	0.03797	0.02982	-0.00815
29(C)	0.034	0.02894	-0.00506
30(C)	0.03699	0.02874	-0.00825
31(C)	0.03503	0.02975	-0.00528
32(C)	0.03629	0.02808	-0.00821
33(C)	0.00816	0.00556	-0.0026
34(C)	0.0082	0.00545	-0.00275
35(H)	0.0035	0.00612	0.00262
36(H)	0.00343	0.00632	0.0029
37(H)	0.00337	0.00582	0.00245
38(H)	0.00376	0.00676	0.00300
39(H)	0.00331	0.00622	0.00291
40(H)	0.00143	0.00286	0.00143
41(H)	0.00176	0.00332	0.00156
42(H)	0.00216	0.00339	0.00123
43(H)	0.00217	0.0034	0.00122
44(H)	0.00143	0.00284	0.0014
45(H)	0.00178	0.00329	0.00152
46(H)	0.00283	0.00647	0.00364
47(H)	0.00271	0.0072	0.0045
48(H)	0.00256	0.00762	0.00506
49(H)	0.00326	0.00731	0.00405

50(H)	0.0027	0.00723	0.00453
51(H)	0.00282	0.00648	0.00366
52(H)	0.00325	0.00732	0.00407
53(H)	0.00255	0.00765	0.00510
54(H)	0.00378	0.00671	0.00293
55(H)	0.00332	0.00617	0.00285
56(H)	0.00351	0.00609	0.00257
57(H)	0.00344	0.00631	0.00287
58(H)	0.00338	0.00581	0.00243
59(H)	0.00214	0.00339	0.00124
60(H)	0.00142	0.00288	0.00146
61(H)	0.00175	0.00333	0.00158
62(H)	0.00143	0.00284	0.00141
63(H)	0.00177	0.00328	0.00151
64(H)	0.00218	0.00341	0.00123

Table S4. Orbital-weighted Fukui functions and orbital-weighted dual descriptors of Na^+ and each atom in the PC molecule in System 2.

Atom index	f_w^-	f_w^+	Δf_w
1(C)	0.04016	0.02205	-0.01811
2(C)	0.03837	0.02281	-0.01556
3(C)	0.03947	0.02161	-0.01786
4(C)	0.04045	0.02199	-0.01845
5(C)	0.04102	0.02321	-0.0178
6(C)	0.03733	0.02218	-0.01514
7(C)	0.01237	0.0067	-0.00567
8(C)	0.02724	0.03314	0.00591
9(C)	0.00799	0.00732	-0.00067
10(C)	0.00865	0.00447	-0.00418
11(C)	0.02364	0.03564	0.012
12(C)	0.02202	0.03694	0.01492
13(C)	0.02327	0.03366	0.01039
14(C)	0.02119	0.03625	0.01506
15(C)	0.02474	0.03732	0.01258
16(O)	0.00806	0.01306	0.00501
17(C)	0.00425	0.03256	0.02831
18(O)	0.01015	0.01207	0.00192
19(O)	0.00883	0.02038	0.01155
20(C)	0.03204	0.02556	-0.00648
21(C)	0.03133	0.02779	-0.00354
22(C)	0.03314	0.02602	-0.00712
23(C)	0.03685	0.02454	-0.01231

24(C)	0.03419	0.02773	-0.00646
25(C)	0.03048	0.02764	-0.00284
26(C)	0.01431	0.00553	-0.00877
27(C)	0.04215	0.02122	-0.02093
28(C)	0.04232	0.0229	-0.01942
29(C)	0.03812	0.02213	-0.01599
30(C)	0.04116	0.02159	-0.01957
31(C)	0.03923	0.02228	-0.01694
32(C)	0.0407	0.021	-0.01971
33(C)	0.00935	0.00402	-0.00533
34(C)	0.0095	0.00389	-0.00562
35(H)	0.00386	0.00443	0.00057
36(H)	0.00382	0.00461	0.00079
37(H)	0.00373	0.00443	0.0007
38(H)	0.00397	0.00542	0.00145
39(H)	0.0037	0.00452	0.00082
40(H)	0.00136	0.00639	0.00503
41(H)	0.00176	0.00499	0.00323
42(H)	0.00211	0.00306	0.00095
43(H)	0.00232	0.00367	0.00135
44(H)	0.00155	0.00206	0.00051
45(H)	0.00188	0.00254	0.00066
46(H)	0.00214	0.00742	0.00527
47(H)	0.00192	0.00945	0.00752
48(H)	0.00184	0.00941	0.00757
49(H)	0.00267	0.00802	0.00535

50(H)	0.00278	0.00632	0.00354
51(H)	0.00302	0.00495	0.00193
52(H)	0.00345	0.00587	0.00242
53(H)	0.00263	0.00765	0.00502
54(H)	0.00424	0.00585	0.00161
55(H)	0.0038	0.00524	0.00145
56(H)	0.00397	0.0044	0.00043
57(H)	0.00393	0.00451	0.00058
58(H)	0.00386	0.00416	0.0003
59(H)	0.00246	0.00234	-0.00012
60(H)	0.00169	0.00198	0.00029
61(H)	0.00208	0.00233	0.00025
62(H)	0.00171	0.00192	0.0002
63(H)	0.0021	0.00232	0.00022
64(H)	0.00254	0.00238	-0.00016
65(Na)	0.003	0.11026	0.10726

Table S5. Orbital-weighted Fukui functions and orbital-weighted dual descriptors of K^+ and each atom in the PC molecule.

Atom index	f_w^-	f_w^+	Δf_w
1(C)	0.03943	0.02253	-0.0169
2(C)	0.03765	0.02332	-0.01432
3(C)	0.03867	0.02205	-0.01662
4(C)	0.03968	0.02233	-0.01735
5(C)	0.04029	0.02361	-0.01668
6(C)	0.03657	0.02266	-0.01391
7(C)	0.01217	0.00667	-0.00551
8(C)	0.02882	0.03126	0.00245
9(C)	0.00766	0.00768	0.00001
10(C)	0.00841	0.00454	-0.00387
11(C)	0.02519	0.03354	0.00835
12(C)	0.0239	0.03463	0.01073
13(C)	0.02517	0.03153	0.00637
14(C)	0.02303	0.03405	0.01102
15(C)	0.02634	0.03505	0.0087
16(O)	0.00891	0.01203	0.00313
17(C)	0.00461	0.02982	0.02521
18(O)	0.0106	0.01123	0.00063
19(O)	0.01056	0.01824	0.00768
20(C)	0.03192	0.0255	-0.00643
21(C)	0.03112	0.02773	-0.00339
22(C)	0.0328	0.02624	-0.00656
23(C)	0.03655	0.02471	-0.01184

24(C)	0.03384	0.02793	-0.00591
25(C)	0.03026	0.0276	-0.00266
26(C)	0.01398	0.00562	-0.00835
27(C)	0.04137	0.02169	-0.01968
28(C)	0.04151	0.0234	-0.01811
29(C)	0.03732	0.02276	-0.01457
30(C)	0.04039	0.02215	-0.01824
31(C)	0.03845	0.02281	-0.01564
32(C)	0.03989	0.0215	-0.01839
33(C)	0.0091	0.00411	-0.00498
34(C)	0.00924	0.00401	-0.00523
35(H)	0.00378	0.00456	0.00079
36(H)	0.00373	0.00476	0.00103
37(H)	0.00363	0.00461	0.00098
38(H)	0.00389	0.0054	0.00151
39(H)	0.00361	0.00465	0.00104
40(H)	0.00123	0.00627	0.00504
41(H)	0.00164	0.00504	0.0034
42(H)	0.00205	0.00332	0.00127
43(H)	0.00225	0.00348	0.00123
44(H)	0.00153	0.00217	0.00065
45(H)	0.00181	0.00259	0.00078
46(H)	0.00228	0.00686	0.00458
47(H)	0.00208	0.00821	0.00613
48(H)	0.00199	0.00829	0.00629
49(H)	0.00284	0.00749	0.00465

50(H)	0.00275	0.00615	0.00339
51(H)	0.00299	0.00505	0.00206
52(H)	0.00341	0.00595	0.00255
53(H)	0.00261	0.00714	0.00453
54(H)	0.00414	0.00592	0.00177
55(H)	0.0037	0.00568	0.00198
56(H)	0.00388	0.0046	0.00072
57(H)	0.00383	0.00466	0.00082
58(H)	0.00377	0.00429	0.00052
59(H)	0.00239	0.00241	0.00002
60(H)	0.00164	0.00205	0.00041
61(H)	0.00201	0.00241	0.0004
62(H)	0.00165	0.00199	0.00034
63(H)	0.00204	0.00241	0.00037
64(H)	0.00247	0.00246	-0.00001
65(K)	0.00291	0.12468	0.12177

References

1 F. Neese, *Mol. Sci.*, 2022, **12**, e1606.

2 T. Lu and F. Chen, *J. Comput. Chem.*, 2012, **33**, 580-592.

3 K. G. Sprenger, V. W. Jaeger and J. Pfaendtner, *J. Phys. Chem. B*, 2015, **119**, 5882-5895.

4 Y. Qiu, Y. Jiang, Y. Zhang and H. Zhang, *J. Phys. Chem. B*, 2021, **125**, 13502-13518.

5 M. J. Abraham, T. Murtola, R. Schulz, S. Páll, J. C. Smith, B. Hess and E. Lindahl, *SoftwareX*, 2015, **1-2**, 19-25.

6 W. Humphrey, A. Dalke and K. Schulten, *J. Mol. Graph.*, 1996, **14**, 33-38.

7 M. J. Frisch, G. W. Trucks, H. B. Schlegel, G. E. Scuseria, M. A. Robb, J. R. Cheeseman, G. Scalmani, V. Barone, G. A. Petersson, H. Nakatsuji, et al., Gaussian 16, Gaussian, Inc., Wallingford, CT, 2016.

8 A. D. Becke, *Phys. Rev. A*, 1988, **38**, 3098-3100.

9 A. D. Becke, *J. Chem. Phys.*, 1993, **98**, 5648-5652.

10 C. Lee, W. Yang and R. G. Parr, *Phys. Rev. B*, 1988, **37**, 785-789.

11 F. Weigend and R. Ahlrichs, *Phys. Chem. Chem. Phys*., 2005, **7**, 3297-3305.

12 K. A. Peterson, D. Figgen, E. Goll, H. Stoll and M. Dolg, *J. Chem. Phys.*, 2003, **119**, 11113-11123.

13 J. Zheng, X. Xu and D. G. Truhlar, *Theor. Chem. Acc.*, 2011, **128**, 295-305.
